# Mito-priming as a method to engineer Bcl-2 addiction

**DOI:** 10.1038/ncomms10538

**Published:** 2016-02-02

**Authors:** Jonathan Lopez, Margaux Bessou, Joel S. Riley, Evangelos Giampazolias, Franziska Todt, Tony Rochegüe, Andrew Oberst, Douglas R. Green, Frank Edlich, Gabriel Ichim, Stephen W. G. Tait

**Affiliations:** 1 Cancer Research UK Beatson Institute, Switchback Road, Glasgow G61 1BD, UK; 2 Institute of Cancer Sciences, University of Glasgow, Garscube Estate, Switchback Road, Glasgow G61 1BD, UK; 3 Institute for Biochemistry and Molecular Biology, ZBMZ, Faculty of Medicine, University of Freiburg, 79104 Freiburg, Germany; 4 Department of Immunology, University of Washington, Campus Box 358059, 750 Republican Street, Seattle, Washington, USA; 5 Department of Immunology, St Jude Children's Research Hospital, 262 Danny Thomas Place, Memphis, Tennessee 38105, USA; 6 BIOSS, Centre for Biological Signaling Studies, University of Freiburg, 79104 Freiburg, Germany

## Abstract

Most apoptotic stimuli require mitochondrial outer membrane permeabilization (MOMP) in order to execute cell death. As such, MOMP is subject to tight control by Bcl-2 family proteins. We have developed a powerful new technique to investigate Bcl-2-mediated regulation of MOMP. This method, called mito-priming, uses co-expression of pro- and anti-apoptotic Bcl-2 proteins to engineer Bcl-2 addiction. On addition of Bcl-2 targeting BH3 mimetics, mito-primed cells undergo apoptosis in a rapid and synchronous manner. Using this method we have comprehensively surveyed the efficacy of BH3 mimetic compounds, identifying potent and specific MCL-1 inhibitors. Furthermore, by combining different pro- and anti-apoptotic Bcl-2 pairings together with CRISPR/Cas9-based genome editing, we find that tBID and PUMA can preferentially kill in a BAK-dependent manner. In summary, mito-priming represents a facile and robust means to trigger mitochondrial apoptosis.

## Abstract (web summary)

Apoptosis often requires mitochondrial outer membrane permeabilization, a process targeted by Bcl-2-binding BH3 mimetics. Here the authors describe and apply 'mito-priming', a method that allows triggering mitochondrial apoptosis in a synchronous manner, facilitating the investigation of mitochondrial apoptosis and its regulation by Bcl-2 proteins.

## Graphical abstract

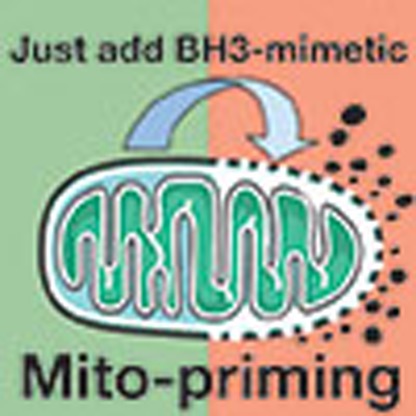

Following most apoptotic stimuli, mitochondrial outer membrane permeabilization (MOMP) is essential for cell death. MOMP leads to the release of mitochondrial intermembrane space proteins such as cytochrome *c* that activate caspase proteases causing rapid cell death^1^. However, even in the absence of caspase activity, MOMP typically kills cells and therefore represents a point-of-no-return^2^. Given this pivotal role in dictating life and death, MOMP is highly regulated, primarily by pro- and anti-apoptotic members of the Bcl-2 protein family^3^.

Evasion from apoptosis is considered a hallmark of cancer^4^. Paradoxically, while apoptotic inhibition promotes cancer, tumour cells often display increased apoptotic sensitivity relative to normal tissue. Underlying this increased sensitivity, are altered levels of pro- and anti-apoptotic Bcl-2 proteins. Due to the pro-apoptotic stresses that cancer cells encounter, anti-apoptotic Bcl-2 function is required for cell survival to counteract pro-apoptotic BH3-only protein function. Cancer cells in this state are termed ‘primed-to-die' and are sensitive to apoptosis-inducing therapies^5^,^6^. Importantly, targeted anti-cancer therapies called BH3 mimetics have recently been developed to exploit this Bcl-2 dependency. In a manner similar to BH3-only proteins, BH3 mimetics bind to and inhibit anti-apoptotic Bcl-2 function^7^.

Due to the wide-ranging roles of apoptosis in health and disease, the regulation of MOMP by Bcl-2 proteins has been intensively studied. Nevertheless, methods to investigate mitochondrial apoptosis are complicated by commonly used treatments, such as staurosporine, that induce MOMP over many hours, in an asynchronous manner and often with off-target, non-MOMP-dependent effects. Current methods to ‘cleanly' induce mitochondrial apoptosis include ER/tamoxifen or doxycycline-based induction of BH3-only protein activity^8^,^9^,^10^,^11^. However, these approaches remain far from ideal due to various factors that include low potency, lack of general applicability, extended time of induction and, in some cases, direct effects of the chemical inducer on mitochondrial function^12^. Circumventing these problems, our aim was to develop a technique that would rapidly and synchronously induce apoptosis over a cellular population in an effective manner. Ideally, such an approach would be applicable to any cell of choice. A second criterion was that any technique should also permit investigation of Bcl-2-mediated regulation of MOMP, for example allowing investigation of BH3-only protein specificity for the executioner proteins BAX or BAK.

With these goals in mind, we chose to mimic primed-to-die cancer cells through a method we call ‘mito-priming'. We reasoned that co-expression of pro- and anti-apoptotic Bcl-2 proteins should render cells highly sensitive to the addition of BH3 mimetic compounds (Fig. 1a). Bcl-2/BH3-only protein complexes are highly dynamic; therefore, we predicted that addition of BH3 mimetics that compete for Bcl-2 binding would free sufficient BH3-only proteins to activate BAX/BAK, leading to MOMP and to cell death. Our expectation was that mito-priming should permit engineering of Bcl-2 addiction to any cell-type. Indeed, we find that mito-priming is a potent and generally applicable method to induce mitochondrial apoptosis in a clean and controllable manner. Furthermore, mito-priming represents a robust means of interrogating functional relationships within the Bcl-2 family network. We highlight the utility of this method to compare the potency and selectivity of available BH3 mimetics and to identify selective requirements for the proapoptotic effectors BAX and BAK in BH3-only protein driven apoptosis.

## Results

### Mito-priming permits rapid induction of apoptosis

**Figure 1:**
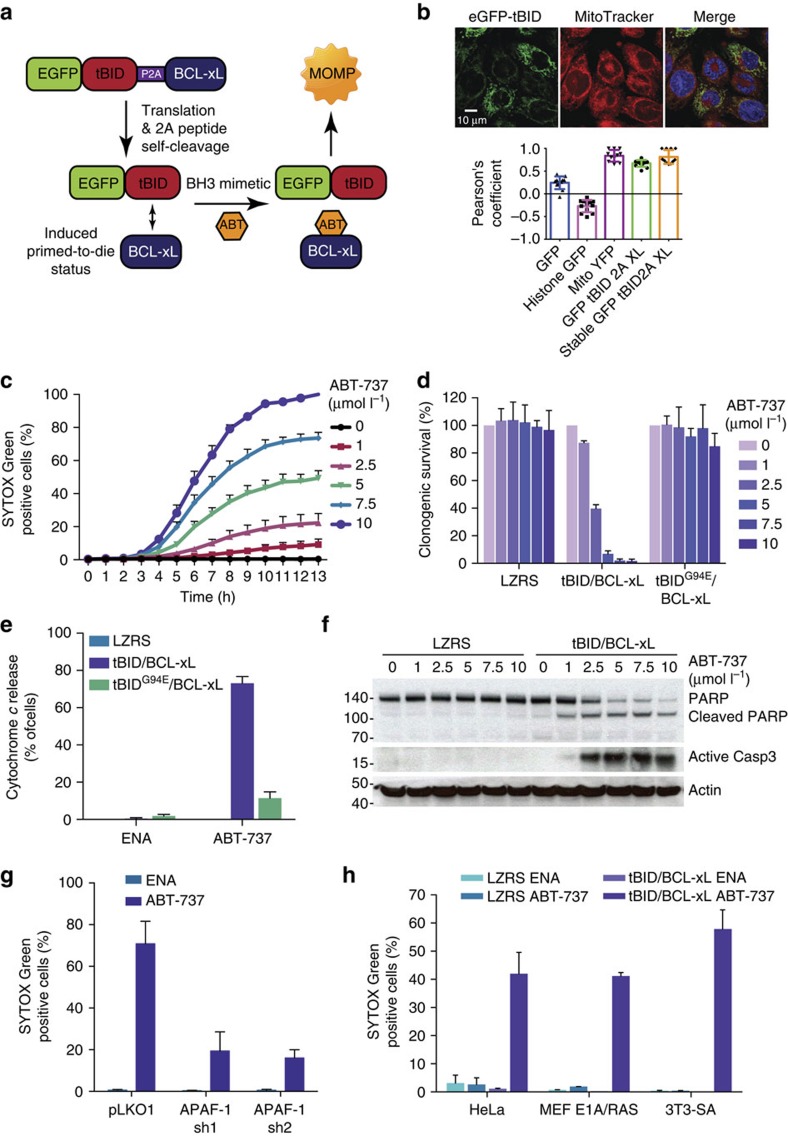
Mito-priming as a method of induced Bcl-2 addiction. (**a**) Method outline. (**b**) SVEC cells expressing eGFP-tBID 2A BCL-xL or other constructs were co-stained with MitoTracker Deep Red. Colocalization was quantified using the Pearson's coefficient. Representative images show eGFP-tBID expression and MitoTracker Deep Red staining from SVEC cells stably expressing eGFP-tBID 2A BCL-xL. Scale bar, 10 μm. (**c**) SVEC cells expressing eGFP-tBID 2A BCL-xL were treated with ABT-737 and analysed for cell viability using an IncuCyte imager and SYTOX Green exclusion. Percentage cell death was calculated by normalizing against maximal cell death (13-h treatment with 10 μmol l^−1^ ABT-737). Error bars represent the standard error of the mean (s.e.m.) from three independent experiments. (**d**) SVEC cells stably expressing the indicated constructs were treated with increasing concentrations of ABT-737 (μmol l^−1^; 24 h) and assayed for clonogenic survival. Error bars represent the s.d. of triplicate samples from a representative experiment carried out twice independently. (**e**) SVEC cells stably expressing indicated constructs were treated with increasing concentration of ABT-737 (μmol l^−1^) for 6 h with 10 μmol l^−1^ ABT-737 in presence of Q-VD-OPh. Cells were quantified for cytochrome *c* release by confocal microscopy. Error bars represent the s.e.m. of three independent experiments. (**f**) SVEC cells stably expressing the indicated constructs were treated for 6 h with ABT-737 (μmol l^−1^). PARP and caspase-3 cleavage was determined by western blot. Actin was probed as a loading control. (**g**) SVEC cells stably expressing the indicated constructs were treated for 13 h with 10 μmol l^−1^ ABT-737 or enantiomer (ENA). Cell viability was determined as in **c**. Percentage cell death was calculated by normalizing against 100% cell death (24-h treatment with 1 μmol l^−1^ actinomycin D). (**h**) Cell lines stably expressing control vector (pLKO1) or two independent shRNA sequences targeting APAF-1 were treated for 16 h with enantiomer (ENA) or ABT-737. 100% death: 24-h treatment with 1 μmol l^−1^ actinomycin D (for each line). Cell viability was determined as in **c**. Percentage cell death was calculated by normalizing against 100% cell death (24-h treatment with 1 μmol l^−1^ actinomycin D). In **g**,**h** error bars represent the s.e.m. of three independent experiments. See also Supplementary Fig. 1 and Supplementary Movies 1–3. Q-VD-OPh, quinolyl-valyl-*O*-methylaspartyl-(2,6-difluoro- phenoxy)-methyl ketone.

In the mito-priming method, we predicted that co-expression of pro- and anti-apoptotic Bcl-2 proteins should enforce a Bcl-2 survival dependency on cells (Fig. 1a). To test this, the BH3-only protein tBID (fused to GFP) was expressed together with anti-apoptotic BCL-xL, using a 2A self-cleaving peptide sequence to enable equimolar co-expression^13^. SVEC cells stably expressing GFP–tBID 2A BCL-xL were generated by retroviral transduction and flow-cytometry-based cell sorting. Western blot analysis verified expression of both GFP–tBID and BCL-xL, and confocal microscopy demonstrated mitochondrial localization of GFP–tBID, which was confirmed by a high Pearson's co-efficient of co-localization with Mitotracker Deep Red FM (Fig. 1b, Supplementary Fig. 1a). We next determined the apoptotic sensitivity of GFP–tBID 2A BCL-xL expressing cells following treatment with the prototypic BH3 mimetic, ABT-737 (ref. 14). Cell viability was determined by live-cell imaging and SYTOX Green exclusion (Fig. 1c). Importantly, ABT-737 treatment rapidly induced cell death in GFP–tBID 2A BCL-xL expressing cells in a synchronous and concentration-dependent manner (Fig. 1c, Supplementary Movies 1 and 2). In line with an on-target effect, treatment with ABT-737 alone, but not its less-active enantiomer, caused cell death (Supplementary Fig. 1b). Moreover, BH3 mimetic-induced death was dependent upon active tBID, since ABT-737 treatment of cells expressing an inactive mutant of tBID (G94E)^15^ together with BCL-xL failed to trigger cell death as determined by both long- or short-term survival assays (Fig. 1d, Supplementary Fig. 1c,d). Consistent with engagement of mitochondrial apoptosis, ABT-737 treatment led to mitochondrial cytochrome *c* and SMAC release (as a readout of MOMP) (Fig. 1e, Supplementary Movie 3) and caspase activity (as verified by western blot analysis of PARP cleavage) in a concentration-dependent manner (Fig. 1f). Furthermore, inhibition of caspase function following treatment with the pan-caspase inhibitor quinolyl-valyl-O-methylaspartyl-[2,6-difluorophenoxy]-methyl ketone (Q-VD-OPh) (Supplementary Fig. 1e) or by small-hairpin-RNA-mediated knockdown of APAF-1 effectively suppressed ABT-737-induced cell death, confirming engagement of mitochondrial apoptosis (Fig. 1g and Supplementary Fig. 1f). Finally, we examined the general utility of this method in other cell lines. For this purpose, we generated HeLa, E1A/Ras transformed murine embryonic fibroblasts and 3T3-SA cells stably expressing GFP–tBID 2A BCL-xL (Supplementary Fig. 1g,h). Stably expressing cells were treated with ABT-737 or enantiomer and analysed for cell viability by SYTOX Green exclusion. Following ABT-737 treatment, all three cell types efficiently died in a manner that was dependent on GFP–tBID 2A BCL-XL expression (Fig. 1h). These results demonstrate that mito-priming is an effective way to engineer Bcl-2 addiction *in vitro.* Following BH3 mimetic treatment, it provides an easy and robust way to induce mitochondrial-dependent apoptosis in a rapid and synchronous manner, avoiding off-target effects inherent to commonly used treatments.

### Applying mito-priming to characterize BH3 mimetics

**Figure 2:**
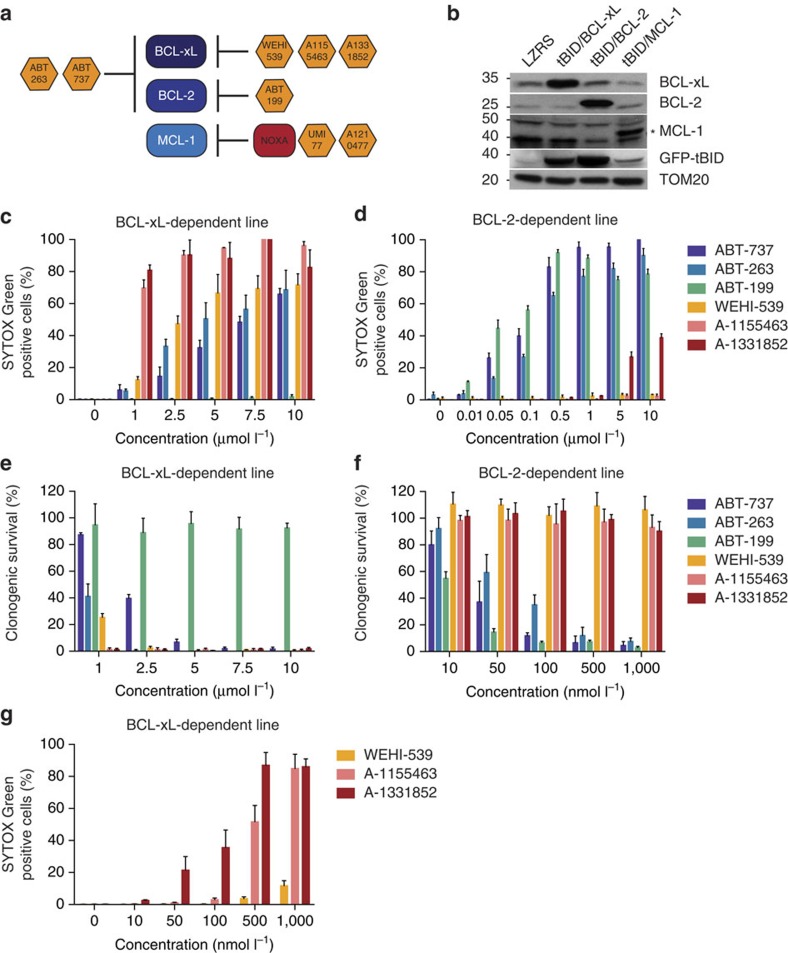
Applying mito-priming to define the selectivity and potency of BH3 mimetics. (**a**) Predicted pattern of inhibition by different BH3 mimetics tested. (**b**) Western blot analysis of SVEC cells stably expressing eGFP-tBID 2A BCL-xL, eGFP-tBID 2A BCL-2 or eGFP-tBID 2A MCL-1. * denotes ectopically expressed MCL-1. (**c**,**d**) SVEC cells stably expressing eGFP-tBID 2A BCL-xL (BCL-xL-dependent line) or eGFP-tBID 2A BCL-2 (BCL2-dependent line) were treated with increasing concentrations of different BH3 mimetics. Cell viability was determined by SYTOX Green dye exclusion and live-cell imaging using an IncuCyte imager. Error bars represent the s.e.m. of three independent experiments. (**e**,**f**) BCL-xL (**e**) or BCL-2-dependent lines (**f**) were treated with increasing concentrations of different BH3 mimetics and analysed for long-term survival by clonogenic assay. Percentage clonogenic survival is plotted relative to untreated cells. Error bars represent the s.d. of triplicate samples from a representative experiment carried out twice independently. (**g**) BCL-xL-dependent line was treated with increasing concentrations of BCL-xL-directed BH3 mimetics. Cell viability was determined by SYTOX Green dye exclusion and live-cell imaging using an IncuCyte imager. Errors bars represent the s.e.m. of three independent experiments. See also Supplementary Fig. 2.

We reasoned that mito-priming would be ideally suited to determine the selectivity and potency of different BH3 mimetics on an identical cellular background. For this purpose, we generated SVEC cells co-expressing GFP-tBID in combination with BCL-2, BCL-xL or MCL-1, hereafter referred to as BCL-2-, MCL-1- or BCL-xL-dependent cells. Mito-primed cells were treated with increasing concentrations of the BCL-2/BCL-xL targeting BH3 mimetics, ABT-737 or ABT-263, the BCL-2 targeting BH3 mimetic ABT-199 or the BCL-xL-directed BH3 mimetics, WEHI-539, A-1155463 or A-1331852 (Fig. 2a,b)^14^,^16^,^17^,^18^,^19^. Cell viability was measured by live-cell imaging and SYTOX Green exclusion. Verifying their on-target selectivity, ABT-737 and −263 efficiently killed both BCL-xL- and BCL-2-dependent cells, ABT-199-induced death specifically in BCL-2-dependent cells and WEHI-539, A-1155463 and A-1331852 specifically killed BCL-xL-dependent cells (Fig. 2c,d). Moreover, none of these BH3 mimetics induced cell death in the MCL-1-dependent SVEC cells (Supplementary Fig. 2a,b). The highly selective cytotoxic effect of all BH3 mimetics was also apparent by long-term clonogenic survival assay (Fig. 2e,f). Notably, supporting previous findings, the dual-BCL-xL/BCL-2 inhibitors ABT-737 and ABT-263 induced death more effectively in BCL-2-dependent cells compared with BCL-xL-dependent cells (Fig. 2c,d)^11^,^20^. Our method also allowed direct comparison of BH3 mimetic potency within a specific Bcl-2-dependent cell line. This revealed that WEHI-539, A-1155463 and A-1331852 and ABT-199 were more effective at inducing death in their cognate target SVEC cells compared to the dual-BCL-2/BCL-xL inhibitors, ABT-263 and ABT-737 (Fig. 2c–f). Furthermore, direct comparison between BCL-xL targeting BH3 mimetics demonstrated that A-1155463 and A-1331852 were significantly more potent than WEHI-539 (Fig. 2c–f). This was further supported by analysing cell viability over an extended concentration range, revealing EC_50_ values of approximately 500 and 100 nmol l^−1^ for A-1155463 and A-1331852 respectively, compared with 2.5 μmol l^−1^ of WEHI-539 (Fig. 2g and Supplementary Fig. 2c). At maximally effective doses, A-1155463, A-1331852 and WEHI-539 displayed high selectivity for BCL-xL whereas at higher levels (5–10 μmol l^−1^) some off-target toxicity for was also observed for A-1331852 in BCL-2-dependent cells (Fig. 2d). These data demonstrate that mito-priming can be used to render cell viability dependent on any given Bcl-2 prosurvival family member. In doing so, this permits rapid analysis of both the potency and selectivity of BH3 mimetic compounds.

### Mito-priming defines potent MCL-1 targeting BH3 mimetics

**Figure 3:**
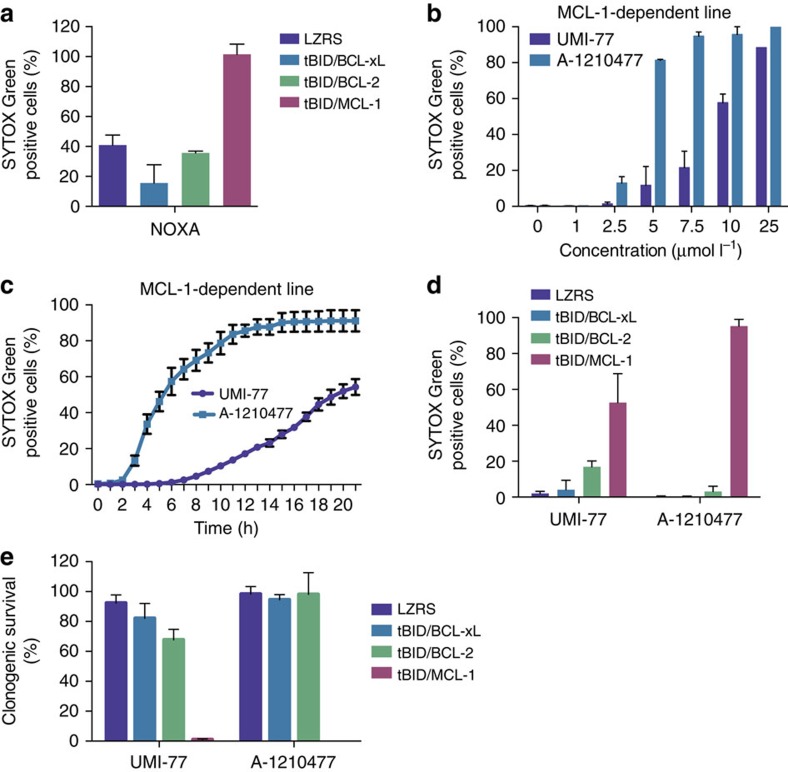
Mito-priming defines potent and specific MCL-1 targeting BH3 mimetics. (**a**) SVEC cells stably expressing FLAG-tBID 2A GFP-BCL-xL, FLAG-tBID 2A GFP-BCL-2 or FLAG-tBID 2A GFP-MCL-1 were transiently transfected with NOXA. Cell viability was analysed 24 h post-transfection by SYTOX Green dye exclusion and live-cell imaging using an IncuCyte imager. Error bars represent the s.e.m. of three independent experiments. (**b**) SVEC cells stably expressing FLAG-tBID 2A GFP-MCL-1 (MCL-1-dependent line) were treated with increasing concentrations of putative MCL-1 inhibitors UMI-77 or A-1210477. Cell viability was analysed 24 h post-treatment by SYTOX Green dye exclusion and live-cell imaging using an IncuCyte imager. Error bars represent the s.e.m. of three independent experiments. (**c**) MCL-1-dependent line was treated with UMI-77 or A-1210477 (both 10 μmol l^−1^) and analysed over time for cell viability by SYTOX Green dye exclusion and live-cell imaging using an IncuCyte imager. Error bars represent the s.e.m. of three independent experiments. (**d**,**e**) SVEC cells stably expressing FLAG-tBID 2A GFP-BCL-xL, FLAG-tBID 2A GFP-BCL-2 or FLAG-tBID 2A GFP-MCL-1 were treated with UMI-77 or A-1210477 (10 μM for 24 h) then cell viability was analysed by SYTOX Green dye exclusion and live-cell imaging using an IncuCyte imager (**d**) or by clonogenic survival assay (**e**). Error bars represent the s.e.m. of three independent experiments for **d** and s.d. of triplicate samples from a representative experiment carried out twice independently for **e**. In all cases, cells were treated with MCL-1 inhibitors in 3% FBS containing DMEM.

Intense interest surrounds the development of MCL-1 inhibitors mainly because current, clinically relevant BH3 mimetics do not target MCL-1. We aimed to use our line stably expressing GFP–tBID 2A MCL-1 to investigate the potency and selectivity of MCL-1 targeted BH3 mimetics. We first confirmed the MCL-1 dependency of these cells by transient expression of NOXA, a BH3-only protein that selectively binds to and inhibits MCL-1 prosurvival function^21^. Accordingly, NOXA expression preferentially killed MCL-1-dependent SVEC cells (Fig. 3a). Next, we investigated the potency of two recently described MCL-1 inhibitors, UMI-77 and A-1210477 (refs 22, 23). MCL-1-dependent SVEC cells were treated with varying doses of each inhibitor and assayed for cell viability by SYTOX Green exclusion and live-cell imaging. Both inhibitors induced cell death in the MCL-1-dependent line in a dose-dependent manner (Fig. 3b). Notably, A-1210477 was significantly more potent than UMI-77, with an EC_50_ below 5 μmol l^−1^ compared with 10 μmol l^−1^ for UMI-77 (Fig. 3b). In line with increased potency, A-1210477 also induced cell death more rapidly (Fig. 3c). The selectivity of these two compounds for targeting MCL-1 over other Bcl-2 family members was next examined. BCL-2-, BCL-xL- and MCL-1-dependent SVEC cells were treated with either inhibitor and assayed for cell viability by short-term, SYTOX Green exclusion and long-term clonogenic survival assay. Both inhibitors preferentially killed MCL-1-dependent SVEC cells, attesting to their on-target specificity (Fig. 3d,e). Whereas A-1210477 solely killed MCL-1-dependent cells, UMI-77 also displayed limited toxicity in BCL-2-dependent SVEC cells (Fig. 3d,e) demonstrating that UMI-77 also affects BCL-2 prosurvival function. Using our approach of induced MCL-1 addiction, our data demonstrate that both UMI-77 and A-1210477 are effective and specific MCL-1 inhibitors, with A-1210477 displaying greater potency and specificity.

### tBID and PUMA can display a dependence on BAK to kill

**Figure 4:**
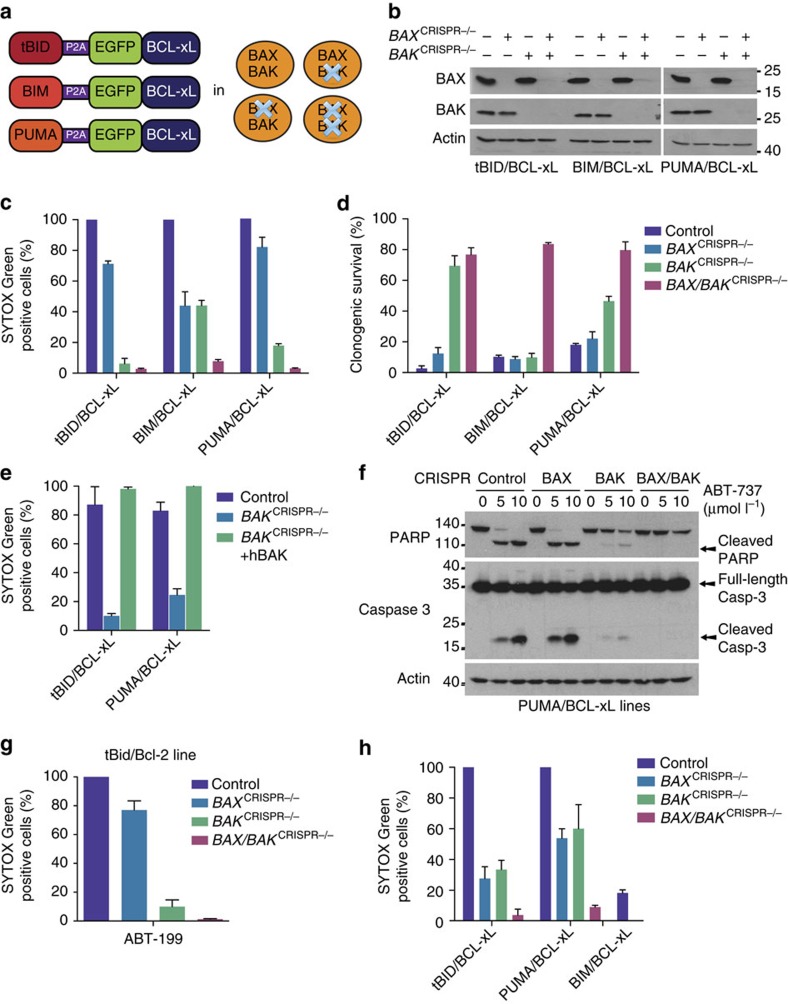
Mito-priming reveals a dependence of tBID and PUMA for BAK over BAX. (**a**) Overview of cell lines generated. (**b**) Western blot analysis of BAX and BAK expression in CRISPR/CAS9 genome edited cell lines. Actin was probed as a loading control. (**c**,**d**) SVEC cells stably expressing tBID-, BIM- or PUMA 2A GFP-BCL-xL were treated with ABT-737 (10 μmol l^−1^) then cell viability was analysed by SYTOX Green dye exclusion and live-cell imaging using an IncuCyte imager (**c**) or by clonogenic survival assay (**d**). In **c** cell death was normalized to treated, empty CRISPR control for each line (Control, each 100% cell death). Error bars represent the s.e.m. of 3 independent experiments (**c**) or s.d. of triplicate samples from a representative experiment carried out twice independently (**d**). (**e**) BAK-deleted or reconstituted SVEC cells stably expressing FLAG-tBID 2A BCL-xL or FLAG-PUMA 2A BCL-xL were treated with ABT-737 (10 μmol l^−1^ for 16 h). Cell viability was analysed by SYTOX Green dye exclusion and IncuCyte imaging. Percentage cell death was calculated following normalization to treated control for each line (each 100% cell death). Errors bars represent the s.e.m. of three independent experiments. (**f**) SVEC cells stably expressing FLAG-PUMA 2A BCL-xL were treated for 4 h with increasing concentrations of ABT-737. Cleavage of caspase-3 and PARP was assessed by western blot. Actin was probed as a loading control. (**g**) Genome-edited SVEC cells stably expressing eGFP-tBID 2A BCL-2 were treated with ABT-199 (10 μmol l^−1^) and analysed for cell viability using SYTOX Green exclusion and IncuCyte imaging. Percentage cell death was calculated following normalization to ABT-199 treated empty CRISPR vector control line for 16 h. Errors bars represent the s.e.m. of three independent experiments. (**h**) Genome-edited HeLa cells stably expressing either eGFP-tBID, BIM or PUMA 2A BCL-xL were treated with 10 μmol l^−1^ ABT-737 for 24 h and analysed for cell viability by SYTOX Green exclusion and IncuCyte imaging. 100% death was set-up as the maximal ABT-737-induced death in the empty control line. Errors bars represent the s.e.m. of three independent experiments. See also Supplementary Fig. 3.

The active form of BID (tBID), BIM and possibly PUMA, represent a subset of BH3-only proteins, called activators, that directly activate the effector proteins BAX and BAK, causing mitochondrial permeabilization and cell death. Recently, tBID and BIM have been shown to display selectivity for BAK over BAX, although whether this also holds true for PUMA remains untested^24^. To investigate this, we applied our approach of mito-priming in *BAX*-, *BAK*- or *BAX/BAK*-deleted cells (Fig. 4a). SVEC cells stably co-expressing tBID, BIM_s_ or PUMA together with BCL-xL were generated (Supplementary Fig. 3a). In stable lines expression levels of PUMA were highest followed by BIM_s_ and tBID. Next, CRISPR-/Cas9-based genome editing was used to delete *BAX* and/or *BAK* (Fig. 4b). Cells were treated with ABT-737 and assayed for cell viability by live-cell imaging and SYTOX Green exclusion or by long-term clonogenic survival assay. As expected, combined deletion of *BAX* and *BAK* prevented cell death in all cases (Fig. 4c,d). Importantly, whereas BIM-induced death displayed no selectivity, specific deletion of *BAK* effectively protected against tBID- and PUMA-induced cell death in both short- and long-term viability assays (Fig. 4c,d). Confirming BAK-dependent selectivity and on-target genome editing, reconstitution of *BAK* CRISPR-edited SVEC cells with BAK restored sensitivity to tBID and PUMA killing, (Fig. 4e and Supplementary Fig. 3b). These data demonstrate that PUMA and tBID selectively require BAK to induce apoptosis in this cell-type. Further supporting this, caspase activity (determined by western blotting for PARP and caspase-3 cleavage), was substantially reduced specifically in BAK-deficient cells following ABT-737 treatment (Fig. 4f). After ABT-737 addition, BCL-xL complexed to either PUMA or BIM_s_ was significantly reduced, as determined by immunopreciptation (Supplementary Fig. 3c). Supporting a transient ‘hit-and-run' interaction, no direct interaction between BIM_s_ or PUMA with BAX or BAK could be detected by immunoprecipitation. To confirm that tBID selectivity for BAK was not due to co-expression of BCL-xL we carried out similar experiments in BCL-2-dependent cells. Using CRISPR/Cas9 genome editing, *BAK*, *BAX* or *BAK* and *BAX* were deleted in GFP–tBID-2A-BCL-2-expressing SVEC cells (Supplementary Fig. 3d). Cells were treated with varying concentrations of ABT-199 and assessed for cell death by SYTOX Green exclusion and live-cell imaging. Importantly, in line with previous results, deletion of *BAK* or *BAX* and *BAK* effectively prevented tBID-induced death, whereas deletion of *BAX* had little effect (Fig. 4g). Finally, we generated HeLa cells expressing GFP-tBID 2A BCL-xL, GFP-PUMA 2A BCL-xL or GFP-BIM_s_ 2A BCL-xL that were deficient in *BAX*, *BAK* or *BAX* and *BAK* and tested their sensitivity to ABT-737 treatment (Fig. 4h, Supplementary Fig. 3e). In contrast to our previous results, deletion of either *BAX* or *BAK* conferred a similar level of protection against tBID and PUMA induced death in HeLa cells, arguing against a selective preference for either BAX or BAK in this setting (Fig. 4h). BAX levels were similar between different cell types ruling this out as a determinant of selectivity (Supplementary Fig. 3f). Interestingly, unlike SVEC cells, HeLa cells expressing GFP-BIM_s_ 2A BCL-xL, poorly responded to ABT-737 treatment (Fig. 4h). Of note, a failure to disrupt BIM/BCL-xL complexes by ABT-737 treatment has also been observed by others^25^. Along these lines, possibly accounting for this resistance, co-immunoprecipitation analysis revealed that ABT-737 treatment failed to disrupt the interaction between BIM_s_ and BCL-xL, (Supplementary Fig. 3g). By combining mito-priming with CRISPR/Cas9-mediated genome editing, our results demonstrate that BH3-only proteins can exhibit a preference for BAK over BAX in some cellular contexts. While previously observed for tBID, we also find a similar preference for BAK by PUMA.

### Mapping determinants of BAK-dependent apoptosis

**Figure 5:**
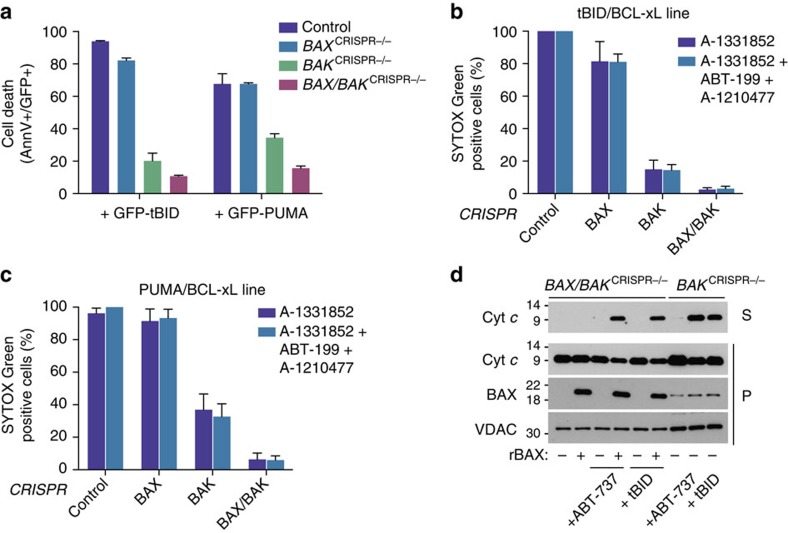
Selective dependence of tBID and PUMA for BAK is independent of prosurvival Bcl-2 function and requires a cellular environment. (**a**) SVEC cell lines were transiently transfected with GFP–tBID or GFP–PUMA. Sixteen hours post transfection the percentage of Annexin V-positive cells in the GFP-expressing population (AnnV+/GFP+) was quantified by flow-cytometry. Error bars represent the s.d. of triplicate samples from a representative experiment carried out twice independently. (**b**) Control, BAX and/or *BAK*^CRISPR*−/−*^ cells stably expressing eGFP-tBID 2A BCL-xL were treated with for 1 h with 10 μmol l^−1^ ABT-199 and A-1210477 before the addition of A-1331852 (10 μmol l^−1^). Cell viability was analysed by SYTOX Green dye exclusion and live-cell imaging using an IncuCyte imager. Percentage cell death was calculated following normalization to treated control for each line (each 100% cell death). Error bars represent the s.e.m. from three independent experiments. (**c**) Control, *BAX* and/or *BAK*^CRISPR*−/−*^ SVEC cells stably expressing eGFP-PUMA 2A BCL-xL were treated as in **b**. Errors bars represent the s.e.m. from three independent experiments. (**d**) Isolated mitochondria from *BAX* and/or *BAK*^CRISPR*−/−*^ SVEC cells stably expressing eGFP-tBid 2A BCL-x_L_ were treated in the presence or absence of 100 nM recombinant wild-type Bax and/or 10 μM ABT-737 or 10 nM recombinant tBID. Mitochondrial permeabilization was monitored by western blot analysis of cytochrome *c* into the supernatant (S). The mitochondrial fraction (P), Bax and VDAC serve as loading controls.

The selective requirement of tBID and PUMA for BAK may be due to a preferential ability to activate BAK or an inability to oppose neutralization of BAX by anti-apoptotic Bcl-2 family members. To discriminate these possibilities, we investigated whether PUMA and tBID also displayed selectivity for BAK in the absence of anti-apoptotic Bcl-2 co-expression. To this end, GFP–tBID and GFP–PUMA were transiently expressed in *BAX* and/or *BAK*-deleted SVEC cells. In line with our earlier findings, tBID and PUMA still preferentially killed in a BAK-dependent manner (Fig. 5a). In a separate approach, we examined whether combined neutralization of MCL-1, BCL-xL and BCL-2 had any effect on the BAK dependency of tBID-induced killing. For this purpose, we used *BAX* and/or *BAK* CRISPR-edited SVEC cells that were stably expressing GFP tBID 2A BCL-xL. Cells were treated with a combination of A-1331852, A-1210477 and ABT-199 to neutralize anti-apoptotic Bcl-2 function and analysed for cell viability by SYTOX Green exclusion and live-cell imaging (Fig. 5b). Importantly, irrespective of pan-Bcl-2 inhibition, tBID-induced cell death still displayed BAK dependence. Similar results were obtained for PUMA-induced cell death (Fig. 5c). Collectively, these data argue that the dependency of tBID and PUMA for BAK is direct and not through differential neutralization of anti-apoptotic Bcl-2 proteins. We next determined whether BH3-only protein selectivity for BAK was an intrinsic property of one or both proteins. To this end, mitochondria were isolated from SVEC cells stably expressing GFP-tBID 2A BCL-xL that were *BAK* or *BAK*/*BAX* deleted. Mitochondria from *BAX*/*BAK*-deleted cells were treated with recombinant BAX in the presence or absence of recombinant tBID or ABT-737. Alternatively, mitochondria from *BAK*-deleted cells were treated with tBID or ABT-737. Mitochondrial permeabilization was assessed by western blotting for cytochrome *c* (Fig. 5d). Importantly, recombinant BAX in combination with tBID or ABT-737 effectively permeabilized mitochondria from *BAX*/*BAK*-deleted cells. Moreover, tBID or ABT-737 treatment effectively permeabilized mitochondria from *BAK*-deleted cells, where residual BAX permitted permeabilization (Fig. 5d, Supplementary Fig. 4). Given that *BAK* deletion effectively prevented tBID-induced killing (Fig. 4c, Supplementary Fig. 3b) these data demonstrate that an intact cellular environment is required for the selective dependence of tBID on BAK. Finally, they argue that the differential requirement for BAX or BAK by specific BH3-only proteins lies at the point of activation of these effectors, as opposed to effects on anti-apoptotic Bcl-2 function.

### BH3-only dependency on BAK is dictated by the BH3-domain

**Figure 6:**
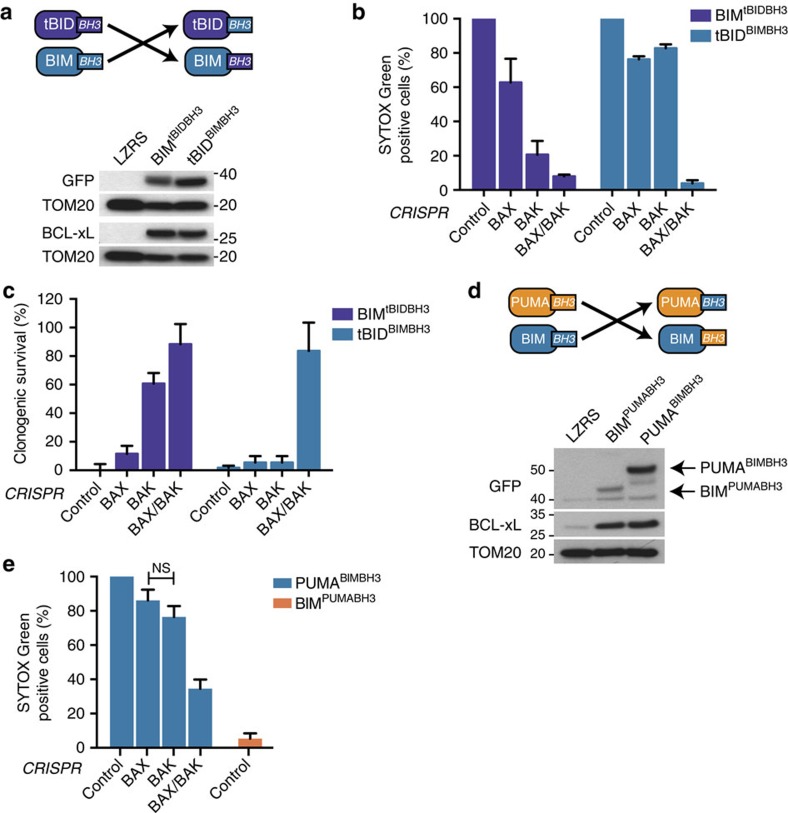
BH3-only protein dependency for BAK is dictated by the BH3-domain. (**a**) SVEC cells stably expressing GFP-BIM^tBID BH3^ or GFP-tBID^BIM BH3^ together with BCL-xL were analysed by Western blot for construct expression. (**b**,**c**) Cell death and long-term clonogenic survival of GFP-BIM^tBID BH3^ and GFP-tBID^BIM BH3^ 2A BCL-xL CRISPR lines treated with 10 μmol l^−1^ ABT-737. (**b**) Error bars represent the s.e.m. of three independent experiments. Percentage cell death was calculated by normalizing to ABT-737-induced death in the empty CRISPR control line (100% death). (**c**) Error bars represent the s.d. of triplicate samples from a representative experiment carried out twice independently. (**d**) SVEC cells stably expressing GFP–BIM^PUMA BH3^ or GFP–PUMA^BIM BH3^ together with BCL-xL were analysed by western blot for construct expression. (**e**) Cell death of GFP–BIM^PUMA BH3^ and GFP–PUMA^BIM BH3^ 2A BCL-xL CRISPR lines treated with 10 μmol l^−1^ ABT-737. Error bars represent the s.e.m. of three independent experiments. Percentage cell death was calculated by normalizing to ABT-737-induced death in the empty CRISPR control line (100% death). See also Supplementary Fig. 4.

We next focused on the BH3-domain as a possible determinant of BAX/BAK specificity. Our earlier results demonstrated that BIM_s_ displayed no selective requirement for BAX or BAK. Therefore, we initially decided to swap the BH3 domains between tBID and BIM_s_ and ask whether this affected BAX/BAK selectivity. SVEC cells stably expressing tBID^BIM BH3^ or BIM^tBID BH3^ together with BCL-xL were generated (Fig. 6a) then *BAX* and/or *BAK* were deleted by CRISPR/Cas9-based genome editing (Supplementary Fig. 5a). Cells were treated with ABT-737 and monitored for cell viability by short-term SYTOX Green exclusion/live-cell imaging or by long-term clonogenic survival assay (Fig. 6b,c). Strikingly, BIM_s_ encoding the BID BH3 domain displayed an absolute dependence on BAK to kill cells. This contrasts with our earlier results demonstrating that wild-type BIM_s_ displayed no selective preference for BAX or BAK (Fig. 4c). Conversely, tBID encoding the BIM BH3 domain lost any specificity, killing *BAK-* or *BAX*-deleted cells equally well. Using a similar approach, we next investigated whether the PUMA BH3 domain also dictated a selective dependence for BAK. SVEC cells stably expressing PUMA^BIM BH3^ or BIM_s_^PUMA BH3^ together with BCL-xL were generated (Fig. 6d) then *BAX* and/or *BAK* were deleted by CRISPR/Cas9-based genome editing (Supplementary Fig. 5b). Mirroring our findings with tBID, engraftment of the BIM BH3-domain into PUMA abolished its selective preference for BAK (Fig. 6e). In the reciprocal swap, cells expressing BIM encoding the PUMA BH3-domain failed to respond to ABT-737 treatment, possibly due to reduced expression of the BIM^PUMABH3^ chimera (Fig. 6d,e). Collectively, these data demonstrate that the BH3-domain is a key determinant in defining selectivity for BAX or BAK.

## Discussion

In this study, we outline an effective means to render cells addicted to anti-apoptotic Bcl-2 prosurvival function—a method we call mito-priming. Following co-expression of pro- and anti-apoptotic Bcl-2 family proteins, cells are highly sensitive to Bcl-2 inhibition allowing BH3 mimetic treatment to trigger apoptosis in a rapid manner. We demonstrate the utility of this method to compare the specificity and potency of different BH3 mimetics. Moreover, using our approach, we find that tBID and PUMA, can display selectivity for BAK over BAX in the activation of mitochondrial apoptosis. Our data demonstrate that tBID and PUMA selectivity for BAK, whilst dictated by the BH3-domain, is independent of anti-apoptotic Bcl-2 inhibition.

The rationale behind mito-priming is based on earlier findings that some cancer cells are ‘primed for death' and require anti-apoptotic Bcl-2 function for survival^5^. Interestingly, ectopic expression of Bcl-2 family members alone can facilitate apoptotic priming in some settings thereby sensitizing cells to pro-death triggers^26^,^27^. Nevertheless, anti-apoptotic Bcl-2 expression often renders cells resistant to apoptotic stimuli. By simultaneously co-expressing pro- and anti-apoptotic Bcl-2 proteins, our method of mito-priming is broadly applicable to different cell types. Furthermore, it allows rapid interrogation of specific BH3-only/Bcl-2 combinations in the regulation of apoptosis. One potential caveat of this method is that mito-priming is subject to modulation by endogenous BH3-only proteins. Given the high-level of exogenous BH3-only expression and the lack of response of the unmodified cell lines used here to BH3 mimetics (thereby indicating low BH3-only load) these effects may be negligible (Fig. 1d–f)^28^. Nevertheless, to circumvent any influence of endogenous BH3-only proteins, mito-priming could be applied in BH3-only protein deficient cells. Finally, the ability to easily generate stably ‘mito-primed' cell lines allows the induction of apoptosis over the whole population in a synchronous manner that is not easily achievable via other means.

Because of their central role in maintaining cell viability, intense interest surrounds the targeting of anti-apoptotic Bcl-2 proteins in cancer. To date, the most effective inhibitors of Bcl-2 function are BH3 mimetics. As a monotherapy, BH3 mimetics effectively kill cancer cells that are so-called ‘primed-to-die'. Due to their high BH3-only protein load, primed-to-die cells require anti-apoptotic Bcl-2 function for survival. By creating a Bcl-2 dependency *in vitro*, our method effectively phenocopies primed-to-die cells such that addition of BH3 mimetic alone leads to rapid cell death. In addition to using BH3 mimetics to engage cell death, our method also provides a rapid and robust means to screen BH3 mimetic potency and selectivity. Using this approach we determined that ABT-199 and A-1331852 are the most potent, currently available inhibitors of BCL-2 and BCL-xL, respectively. Moreover, we identify UMI-77 and A-1210477 as effective inhibitors of MCL-1 function *in vitro*, with A-1210477 displaying the greatest potency and selectivity.

It has recently been shown that tBID and BIM can display selectivity for BAK over BAX in the engagement of MOMP and apoptosis^24^. By combining our approach of induced Bcl-2 addiction with CRISPR/Cas9-based deletion of *BAX* or *BAK*, we find that PUMA, in addition to tBID, can also display selectivity for BAK. Selective dependence for BAK can be uncoupled from BH3-only expression level, since BIM_s_ displayed no selective dependence yet was expressed at levels intermediate to tBID and PUMA. The selective dependence of PUMA and tBID for BAK appears to be independent of differential neutralization of anti-apoptotic Bcl-2 function for two main reasons: (1) selective preference of tBID and PUMA for BAK was maintained following combined neutralization of BCL-2, BCL-xL and MCL-1 (Fig. 5b,c) and (2) BIM, which displays an anti-apoptotic Bcl-2 binding profile similar to tBID and PUMA^5^,^21^,^29^, failed to display any selectivity for BAK or BAX. Nevertheless, our data demonstrate that the BH3-domain is the key determinant for BAK selectivity by tBID and PUMA—engraftment of the tBID BH3-domain into BIM_s_, imparted BAK selectivity on BIM_s_, whereas engraftment of the BIM BH3-domain into tBID and PUMA abolished selectivity (Fig. 6b–e). These findings support previous data demonstrating that BH3-domain peptides alone can display preference for either BAX or BAK^24^. In contrast to these findings, a recent study using liposome and mitochondrial-based permeabilization assays failed to observe BH3-only preferences for BAX or BAK^30^. Why tBID and PUMA display selectivity for BAK is currently unclear although it appears strictly cell-type dependent. In this case of tBID we found that any selective requirement for BAK was completely lost when mitochondrial permeabilization was assayed using isolated mitochondria. This finding, supported by others^30^, argues that the factor(s) mediating selectivity may not solely be integral to differences in Bcl-2 protein interactions.

In summary, by inducing Bcl-2 addiction, mito-priming represents a facile and effective way to induce mitochondrial apoptosis. Importantly, this approach avoids non-MOMP-dependent effects inherent to commonly used apoptotic stimuli. As such, this method should prove an ideal approach to study Bcl-2 regulation of mitochondrial permeabilization, MOMP and its downstream effects. Moreover, mito-priming is well suited for various screening purposes, including the identification and characterization of Bcl-2 targeting compounds as well as novel regulators of mitochondrial-dependent apoptosis.

## Methods

### BH3 mimetics and cell lines

WEHI-539, ABT-199, −263 and −737 were obtained from Chemietek. UMI-77 was obtained from Selleckchem. ABT-737 enantiomer, A-1210477, A-1155463 and A-1331852 were provided by Abbvie Pharmaceuticals. Cell lines were obtained from the ATCC.

### Molecular cloning and cell line generation

2A constructs were directly cloned into LZRS retroviral backbone between EcoRI and XhoI using Gibson Assembly cloning kit (NEB #E5510S). Fragments for the various BH3-only and prosurvival BCL2 members were amplified by PCR using Phusion High Fidelity polymerase (Life Technologies #F530L) using appropriate human cDNAs as template. The P2A sequence used in this work was: 5′ GGATCCGGAGCCACGAACTTCTCTCTGTTAAAGCAAGCAGGAGACGTGGAAGAAAACCCCGGTCCT-3′. Retroviruses were produced using Ampho or Eco Phoenix 293T cells following transfection with Lipofectamine 2000 (Life Technologies). Target cells were infected twice with viral supernatant in the presence of polybrene (1 μg ml^−1^) and then selected for 7 days with Zeocin 200 μg ml^−1^ (Life Technologies #R25001). GFP-positive cells were then sorted on a BD FACSAria cell sorter. For CRISPR/Cas9-mediated deletion of *BAX* and *BAK*, sgRNA sequences were selected using the MIT CRISPR design tool (http://crispr.mit.edu/) and cloned into LentiCRISPRv2 backbone (Addgene #52961). The following guide sequences were used: murine BAX: 5′-CAACTTCAACTGGGGCCGCG-3′(+) and BAK: 5′-GCGCTACGACACAGAGTTCC-3′(−); human BAX: 5′-AGTAGAAAAGGGCGACAACC-3′(−) and BAK: 5′-GCCATGCTGGTAGACGTGTA-3′(−). Lentiviral production, cell infection and selection were performed according to Zhang lab protocol^31^. shRNA sequences targeting murine APAF-1 were GCGGATAAGAAGGTTAAGATT (sh1) and CATGCTTATTTGCACTCTTTA (sh2).

### Viability assays

For short-term viability assays, we used SYTOX Green dye exclusion and live-cell imaging using an IncuCyte FLR imaging system (Essen BioScience). Briefly, 5 × 10^5^ cells were plated into 12-well plates. The following day cells were treated as described in the presence of 30 nM SYTOX Green (Life Technologies). Plates were scanned every hour for 24 h, scanning 4 fields per well. Percentage cell death was calculated by normalizing against maximal cell death following BH3-mimetic treatment. Maximal cell death was calculated bv visual inspection of Incucyte images, where percentage cell death=Sytox Green positive cells/total cells (Sytox Green positive plus negative) × 100. Alternatively, in some experiments, 24-h treatment with 1 μM actinomycin D was used as 100% death control. For cell death analysis by flow-cytometry, cells were stained using Alexa Fluor 647 Annexin V (BioLegend #640912) according to the manufacturer's protocol and quantified on a BD FACSCalibur instrument. For clonogenic survival assay, 800 cells stably expressing the construct of interest were plated into each well of a 6-well tissue culture plate. The following days cells were treated with the indicated stimuli. Ten days later, cells were stained with 1% Methylene Blue in methanol/H_2_O (1:1 vol/vol). Following image capture, colonies were counted automatically using Fiji software (http://fiji.sc/Fiji) and compared with untreated cells (taken as 100% survival). Images were processed as followed: 1/ conversion of scans into 8 bits images and threshold adjustment to remove background; 2/ separation of colonies using the Watershed tool; 3/ counting of colonies using the Analyze Particles tool.

### Microscopy

Confocal imaging was carried out using a Nikon A1R confocal microscope (Nikon Instruments) with laser wavelengths of 405, 488, 561, and 636 nm. Images were acquired with a × 60 NA 1.4 objective. Immunofluorescence staining for cytochrome *c* was performed as follows: SVEC cells stably expressing GFP–tBID 2A BCL-xL and treated with ABT-737 as described in the figure legends were fixed in 4% PFA/PBS for 10 min and permeabilized in 0.2% Triton/PBS for 15 min followed by 1 h blocking in 2% BSA/PBS. The primary antibody and cytochrome *c* (BD Biosciences, 556432, 1/300 in PBS) was incubated overnight. AlexaFluor 647 was used as secondary antibody (Life Technologies, A21245, 1/300 in PBS). Cytochrome *c* was considered released from the mitochondria when it was observed as diffused in the cytosol. For the colocalization studies, SVEC cells stably or transiently expressing GFP–tBID 2A BCL-xL, GFP alone, histone GFP or mito YFP were co-stained with MitoTracker Deep Red FM (ThermoFisher, M22426) according to manufacturer's instruction and then the Pearson's coefficient was determined using ImageJ and JACoP plugin^32^. Cytochrome *c* was considered released from the mitochondria when staining displayed a diffuse localization (as opposed to punctate, mitochondrial localization). Cytochrome *c* release was visually quantified by counting an average of 150 cells (6 different fields) per condition. Error bars represent the s.e.m. of 3 independent experiments.

### Mitochondrial permeabilization assay

Mitochondrial fractions were obtained from cells harvested and centrifuged at 1,200*g* for 5 min at 4 °C. The cell pellet was resuspended in s.e.m. Buffer (10 mmol l^−1^ HEPES, 250 mmol l^−1^ sucrose, pH 7.2) supplemented with protease inhibitors and homogenized. Samples were centrifuged at 500*g* for 3 min at 4 °C. The supernatant was transferred to a new tube and subjected to a centrifugation step at 13,000*g* for 30 min at 4 °C. Sedimented mitochondria were washed two times and incubated in the presence or the absence of 100 nmol l^−1^ recombinant Bax and/or 10 nmol l^−1^ tBid (BD Biosciences) and/or 10 μmol l^−1^ ABT-737 for 1 h at 37 °C. Subsequently, mitochondria and supernatant were separated by centrifugation at 13,000*g* for 5 min. Both fractions were subjected to western blot analysis.

### Immunoprecipitation

Protein lysates were prepared in ice-cold Triton-X 100 lysis buffer (20 mM Tris-Cl (pH 7.4), 135 mM NaCl, 1.5 mM MgCl_2_, 1 mM EGTA, 10% glycerol, 1% Triton-X 100) supplemented with protease inhibitor and PMSF. Lysates (0.5–1 mg) were incubated with 20 μl anti-FLAG M2 affinity gel (A2220, Sigma) for 16 h at 4 °C under end-over-end agitation. Anti-FLAG affinity was then washed five times by centrifugation in ice-cold Triton-X 100 lysis buffer, followed by one wash in TBS (10 mmol l^−1^ Tris-Cl (pH 7.4), 150 mmol l^−1^ NaCl). Bound protein complexes were then eluted in 100 μg ml^−1^ FLAG-peptide (A6002, ApexBio) in TBS for 20 min at room temperature, resuspended in Laemelli sample buffer containing 10 mmol l^−1^ DTT and boiled for 5 min at 95 °C before immunoblot analysis.

### Antibodies

For western blotting the following antibodies were used all at 1:1,000 dilution unless otherwise stated; Cell Signaling: PARP (#9532), APAF-1 (#8723), BAK (D4E4, #12105), and Hsp60 (#4870); from Abcam: active caspase 3 (#ab13847), BCL-xL (#ab32370, used at 1/500 dilution), BCL-2 (#ab18210, used at 1/500 dilution), and Mcl-1 (#ab32087, used at 1/500 dilution); from Santa Cruz: TOM20 (#sc-11415) and BAX (N20, #sc-493); from others: actin (MP Biomedicals #8691001, used at 1/10,000 dilution), Flag M2 (Sigma #F1804) and GFP (in house). For immunofluorescence: cytochrome *c* (BD #556432) was used (1/300 dilution). MitoTracker Deep Red 647 (Life Technologies #M22426) was used at a 100 nM final concentration. Images were acquired on a Nikon A1R confocal instrument.

## Additional information

**How to cite this article:** Lopez, J. *et al.* Mito-priming as a method to engineer Bcl-2 addiction *Nat. Commun.* 7:10538 doi: 10.1038/ncomms10538 (2016).

## Supplementary Material

Supplementary Information Supplementary Figures 1-6

Supplementary Movie 1 SVEC cells stably expressing eGFP-tBID 2A BCL-xL were treated with 10μM ABT-737 and analysed for cell viability by SYTOX Green exclusion and IncuCyte cell imaging every 5 minutes for 21 hours.

Supplementary Movie 2 SVEC cells stably expressing eGFP-tBID 2A BCL-xL were treated with 10μM ABT-737 and analysed for cell viability by Annexin V staining (purple). Images were acquired on a Nikon A1R confocal instrument every 5 minutes for 1.5 hour.

Supplementary Movie 3 SVEC cells stably expressing eGFP-tBID 2A BCL-xL were transfected with SMAC-mCherry. Following treatment with 10μM ABT-737, images were acquired on a Nikon A1R confocal instrument every 5 minutes for 1 hour.
