# Supplementary material for: Mito-priming as a method to engineer Bcl-2 addiction
Source: Nat Commun. 2016 Feb 2;7:10538. doi: 10.1038/ncomms10538 (PMC4740867; doi:10.1038/ncomms10538)
Supplement: Supplementary Information — Supplementary Figures 1-6 [file ncomms10538-s1.pdf]

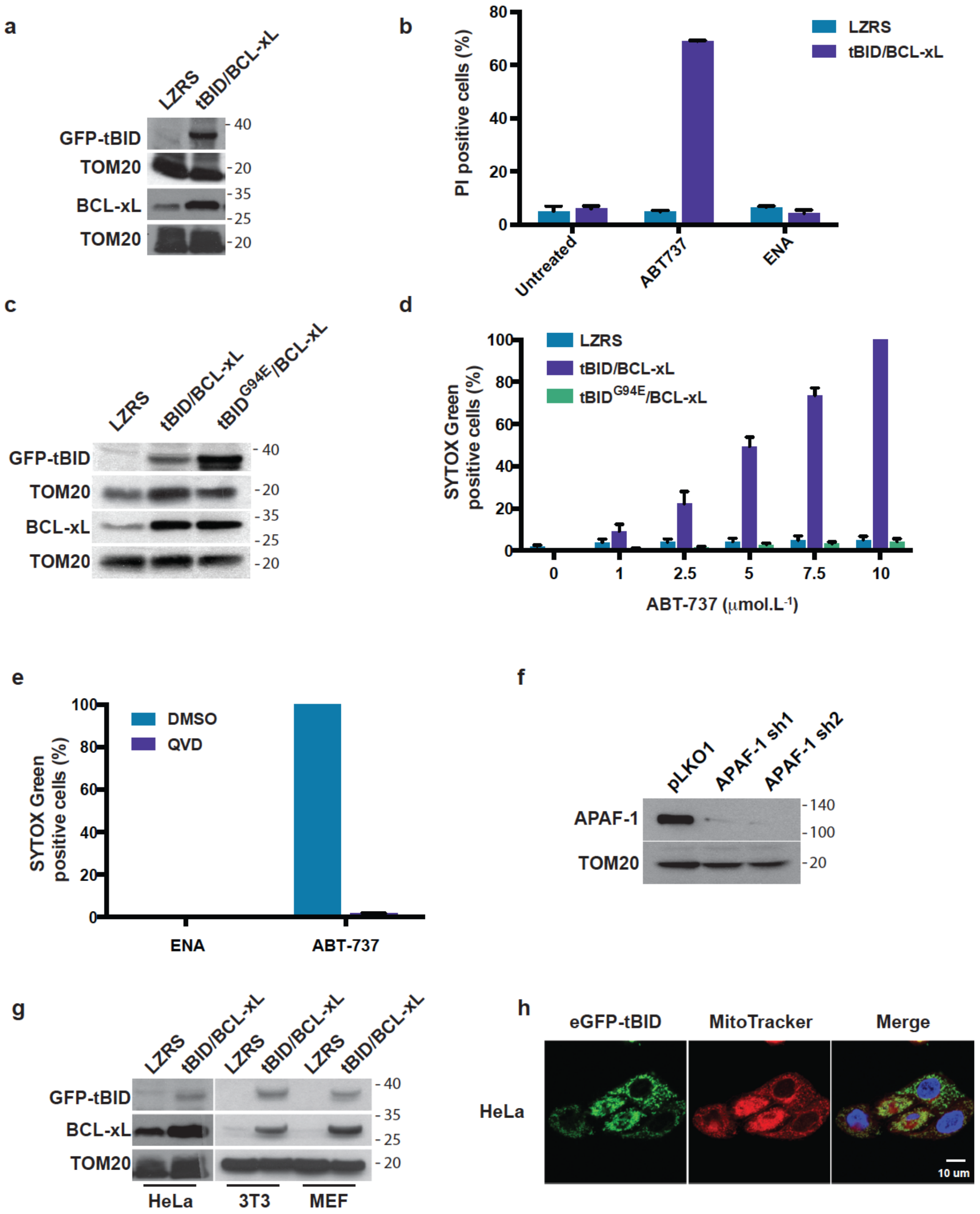

Supplementary Figure 1 (related to Figure 1)

a. SVEC cells stably expressing eGFP-tBID-2A-BCL-xL were analysed by Western blot for expression of eGFP-tBID and BCL-xL. TOM20 was used as a loading control. **b.** SVEC cells stably expressing eGFP-tBID-2A-BCL-xL were treated 16 hours with either 10 µmol.L^{-1} ABT-737 or its inactive enantiomer (ENA) and Propidium iodide positive cells were quantified by flow-cytometry. Error bars represent the SD of triplicate samples from a representative experiment carried out twice independently. **c.** SVEC cells stably

expressing eGFP-tBID-2A-BCL-xL or eGFP-tBID(G94E)-2A-BCL-xL were analysed by Western blot for expression of eGFP-tBID and BCL-xL. TOM20 was used as a loading control. **d.** SVEC cells stably expressing empty vector (LZRS), eGFP-tBID-2A-BCL-xL or eGFP-tBID(G94E)-2A-BCL-xL were treated with increasing concentration of ABT-737 (µmol.L^{-1}). Error bars represent the SEM of 3 independent experiments. **e.** SVEC cells stably expressing eGFP-tBID-2A-BCL-xL were treated with enantiomer (ENA) or ABT-737 in the presence or absence of Q-VD-OPh (30 µmol.L^{-1}), cell viability was measured 8 hours later by SYTOX Green exclusion using an IncuCyte imaging system. 100% cell death was set-up as the maximal ABT-737-induced death in absence of Q-VD-OPh. **f.** SVEC cells stably expressing empty vector eGFP-tBID-2A-BCL-xL together with empty vector or two different shRNA constructs targeting APAF-1 were analysed by Western blot for APAF-1 expression. TOM20 was probed as a loading control. **g.** HeLa, 3T3-SA and MEF E1A/RAS stably expressing LZRS vector or eGFP-tBID-2A-BCL-xL were analysed by Western blot for expression of eGFP-tBID and BCL-xL. TOM20 was probed as a loading control. **h.** HeLa cells stably expressing eGFP-tBID-2A-BCL-xL were stained with MitoTracker Deep Red and analysed by confocal microscopy. Scale bar represents 10 µm.

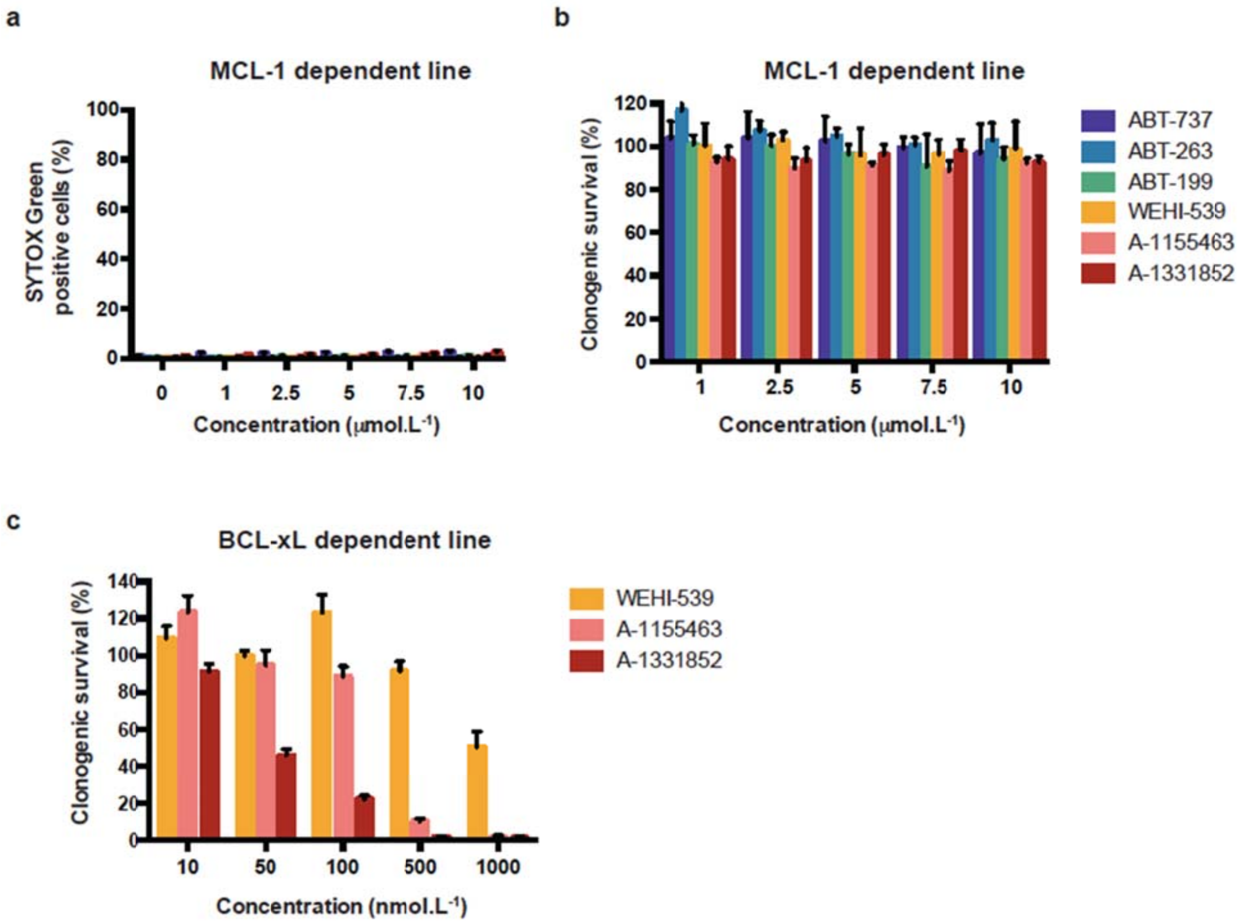

Supplementary Figure 2 (related to Figure 2)

a. SVEC cells stably expressing eGFP-tBID-2A-MCL-1 (MCL-1 dependent line) were treated with increasing concentrations of different BH3 mimetics and analysed for cell viability 24 hours post-treatment using SYTOX Green exclusion and IncuCyte-based cell imaging. Percentage cell death was calculated following normalisation to 24 hours 1 μmol.L^{-1} Actinomycin D treatment. Error bars represent the SEM of 3 independent experiments. **b.** MCL-1 dependent SVEC cells were treated with increasing concentrations of the indicated BH3 mimetics and analysed for long-term viability by clonogenic survival assay. **c.** BCL-xL dependent line was treated with increasing concentrations of the indicated BH3 mimetics and analysed for long-term viability by clonogenic survival assay. For (**b**) and (**c**): Error bars represent the SD of triplicate samples from a representative experiment carried out twice independently. 100% survival: survival in the untreated control.

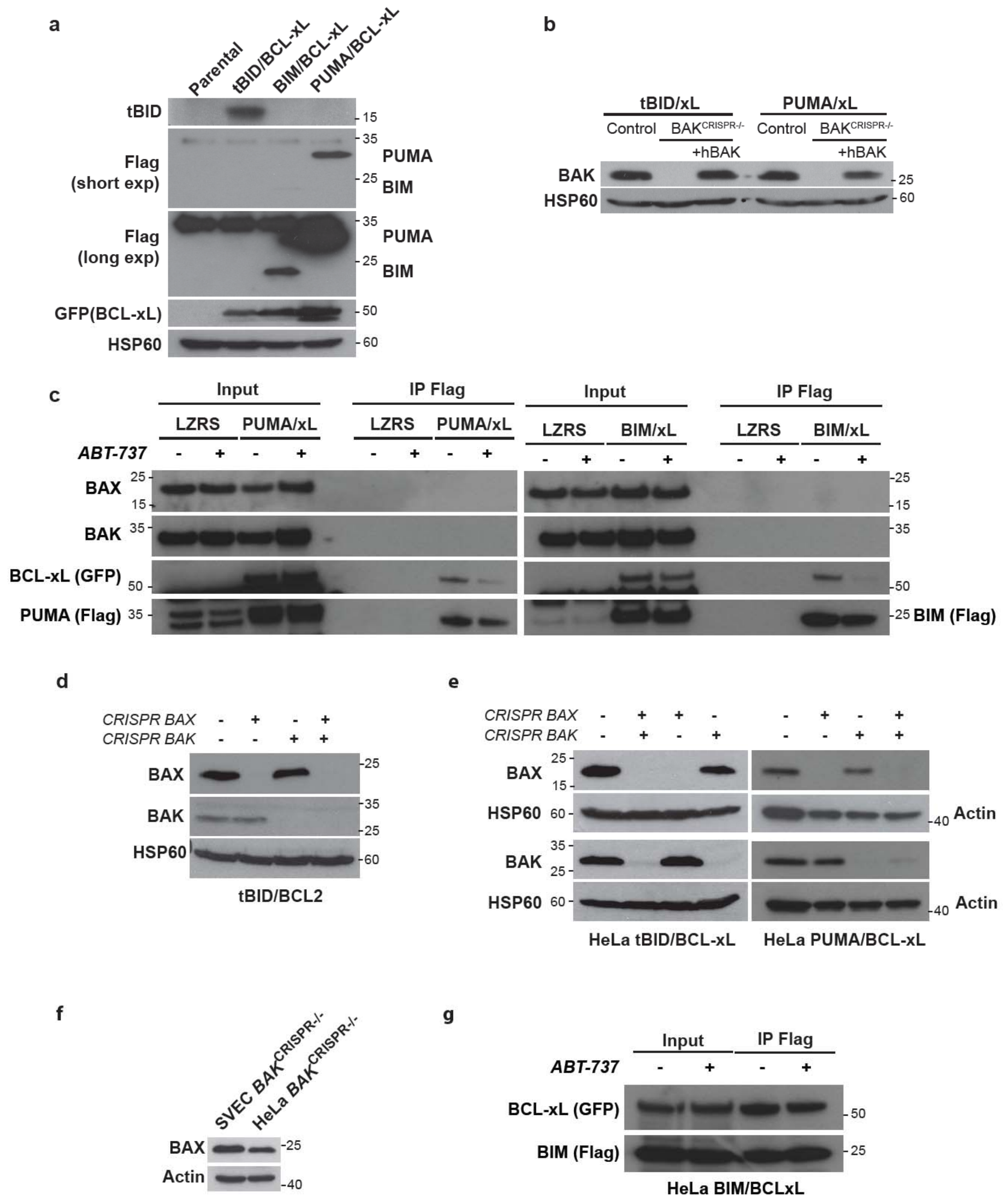

Supplementary Figure 3 (related to Figure 4)

a. SVEC cells stably expressing FLAG-tBID, FLAG-BIM or FLAG-PUMA 2A GFP-BCL-xL were analysed by Western blot for expression of the indicated proteins. Short and long exposures of anti-FLAG blots are shown, due to lack of detection with anti-FLAG antibody, FLAG-tBID was detected with anti-BID antibody. HSP60 was probed as a loading control. **b.** *BAK*$^{CRISPR -/-}$ lines stably expressing tBID or PUMA-

2A-GFP-BCL-xL were transduced with empty vector or vector encoding human BAK. Cells were analysed for BAK expression by Western blot. HSP60 was probed as a loading control. **c.** Flag-PUMA or Flag-BIM were immunoprecipitated from the corresponding mito-primed SVEC lines in presence or absence of ABT-737. Interaction with eGFP-BCL-xL, BAX or BAK was assayed by Western blot. **d.** BCL-2 dependent SVEC cells were transduced with CRISPR vector targeting *BAX* and/or *BAK* were analysed for BAX/BAK expression by Western blot. HSP60 was probed as a loading control. **e.** Control, *BAX*, *BAK* or *BAX/BAK*$^{\text{CRISPR -/-}}$ HeLa cells stably expressing GFP-tBID or GFP-PUMA-2A-BCL-xL were analysed for BAX and BAK expression by Western blot. HSP60 was probed as a loading control. **f.** BAX expression in SVEC and HeLa *BAK*$^{\text{CRISPR-/-}}$ cells was compared by Western blot. Actin was used as a loading control. **g.** Flag-BIM was immunoprecipitated from HeLa BIM/BCL-xL line in presence or absence of ABT-737. Interaction with eGFP-BCL-xL was assayed by Western blot.

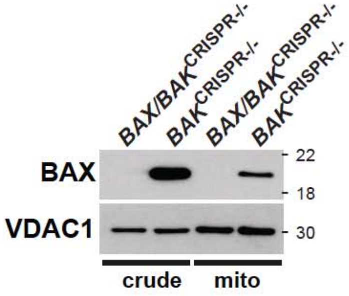

Supplementary Figure 4 (related to Figure 5)

Whole cell lysates (crude) or mitochondria enriched extracts (mito) from BAX/BAK or BAK deleted SVEC cells stably expressing eGFP-tBid-2A-BCL-xL were probed for BAX and VDAC expression by Western blot.

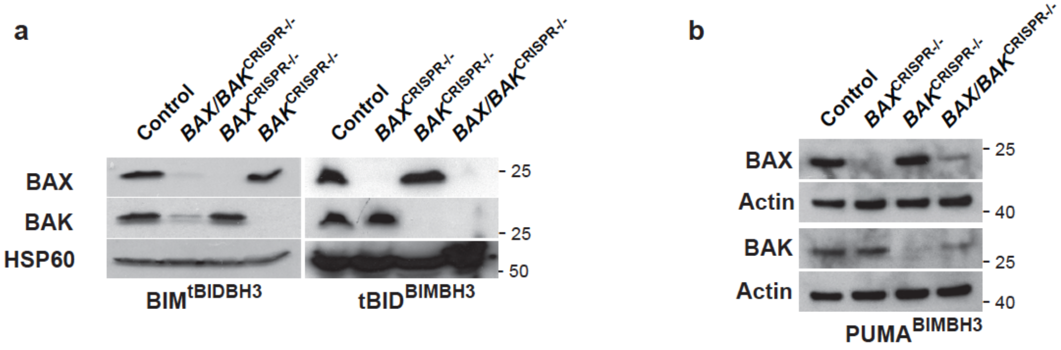

Supplementary Figure 5 (related to Figure 6)

a. Control, *BAX*, *BAK* or *BAX/BAK*$^{\text{CRISPR -/-}}$ SVEC cells stably expressing eGFP-BIM$^{\text{tBID BH3}}$-2A-BCL-xL or eGFP-BID$^{\text{BIM BH3}}$-2A-BCL-xL were analysed by Western blot for BAX and BAK expression. Actin was probed as a loading control**. b.** As in a. but for PUMA/BIM swap.

.

Supplementary Figure 6 (scans)

Fig. 1f

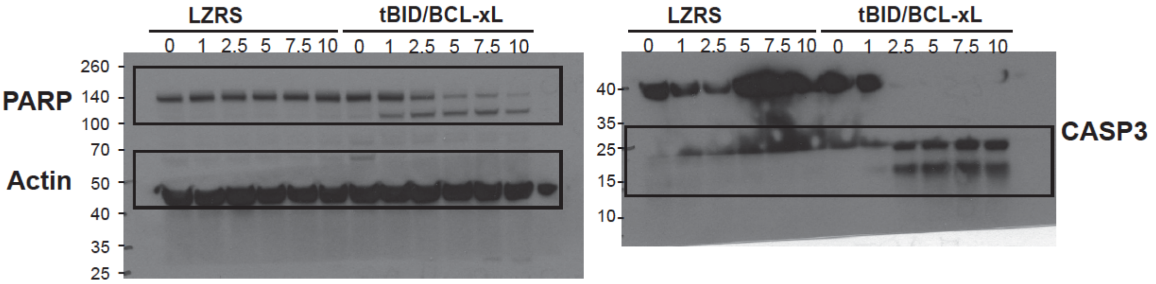

Fig. 2b

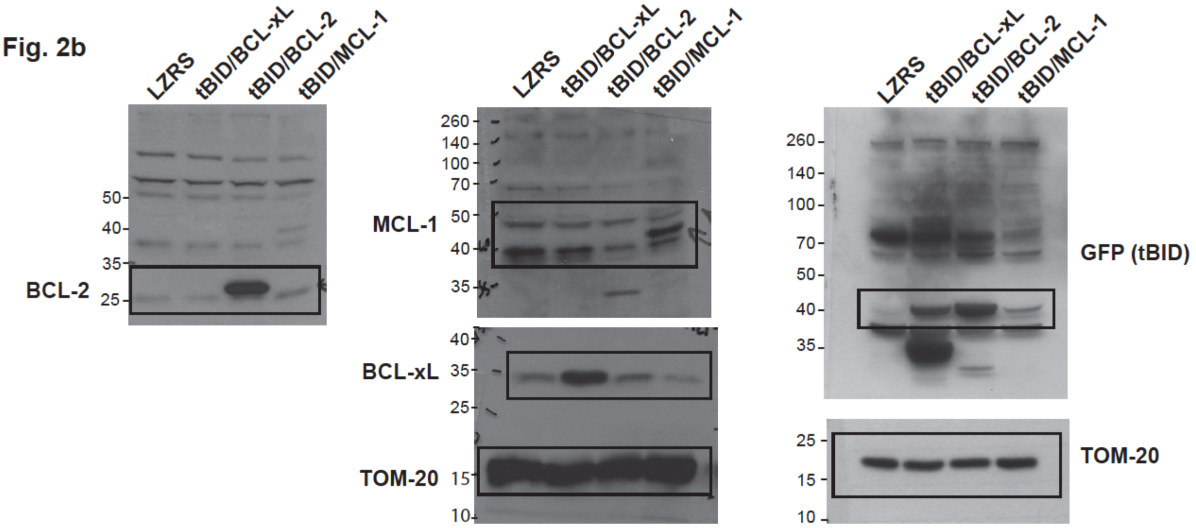

Fig. 4b

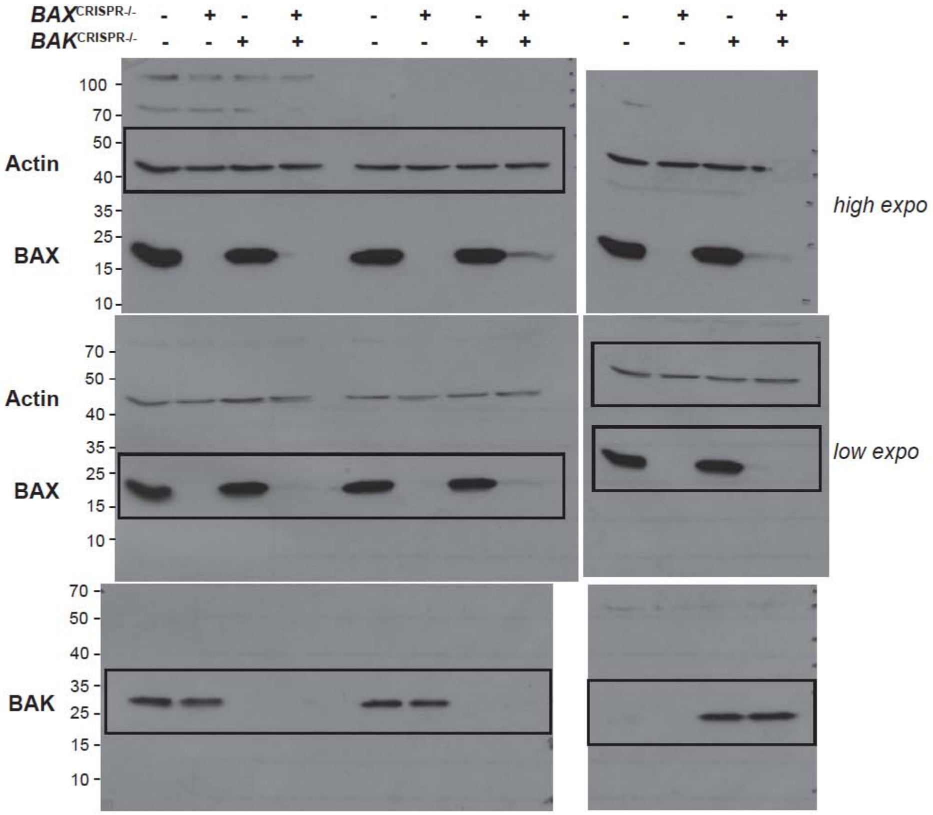
BAX[CRISPR-/-] - + - + - + - + - + - +
BAK[CRISPR-/-] - - + + - - + + - - + +
100
70
50
Actin
40
35
25
BAX
15
10
high expo
70
50
Actin
40
35
25
BAX
15
10
low expo
70
50
40
35
BAK
25
15
10

Fig. 4f

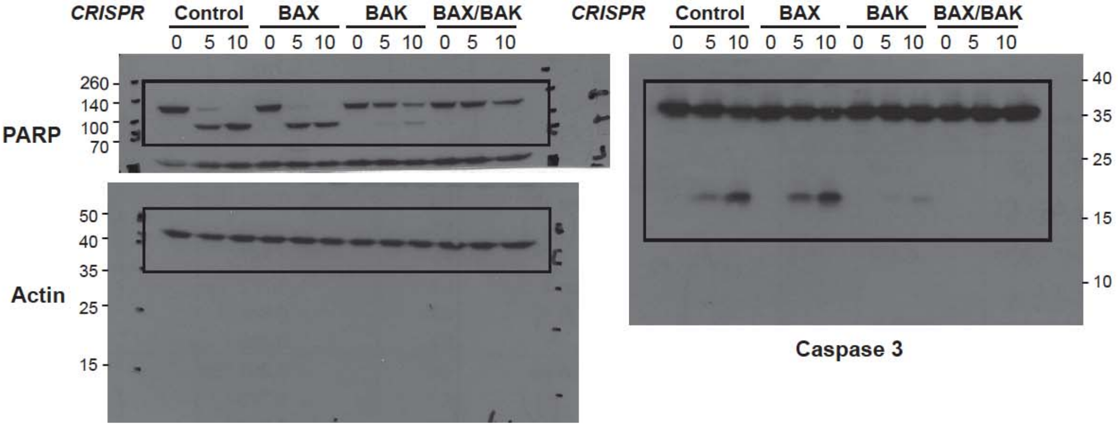

Fig. 5d

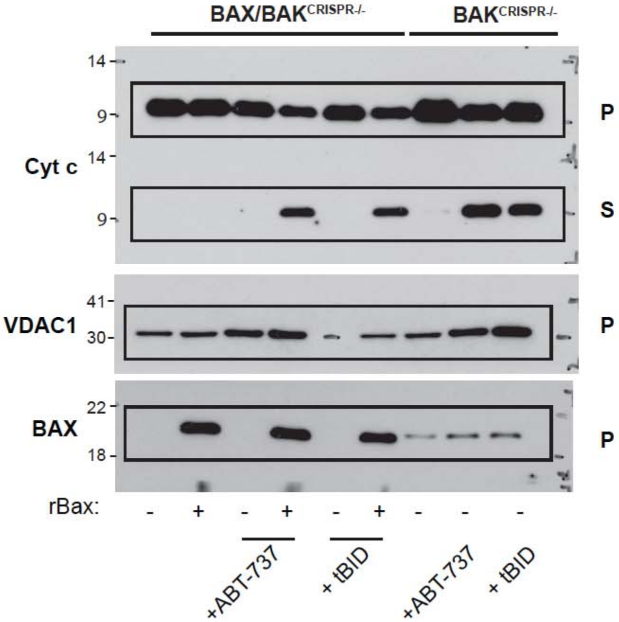

Fig. 6a

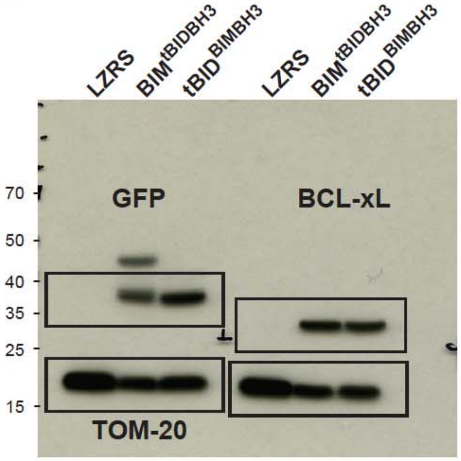

Fig. 6d

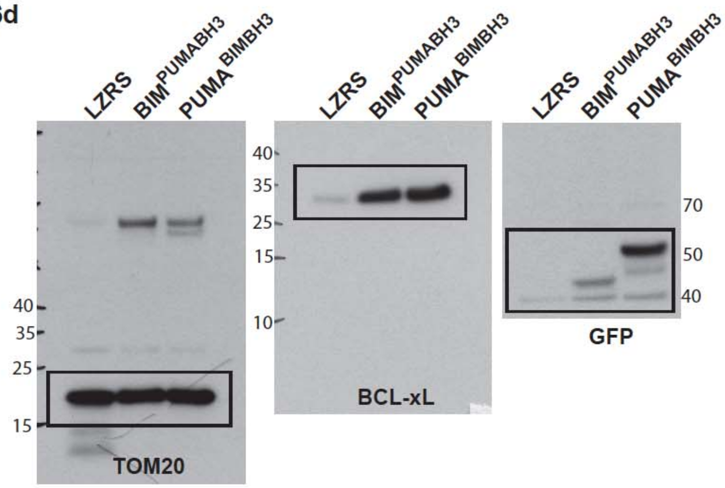

S1a & S1g

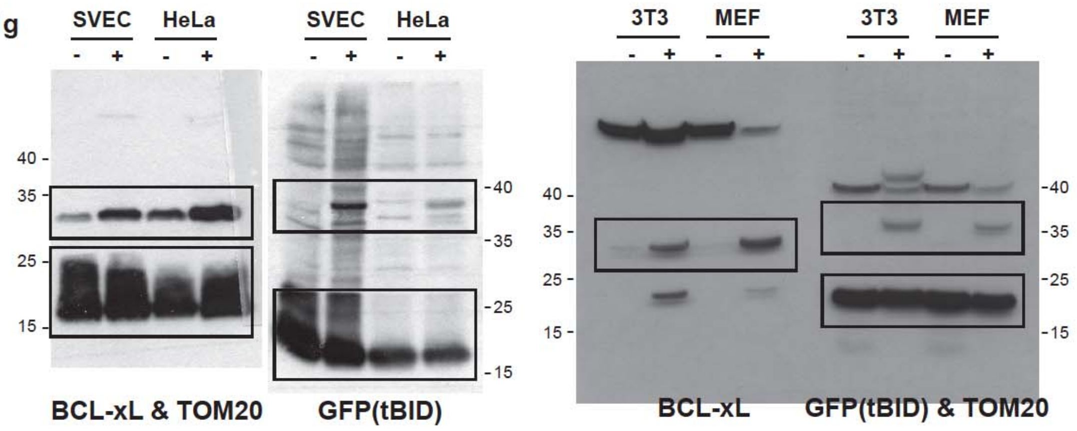

S1c

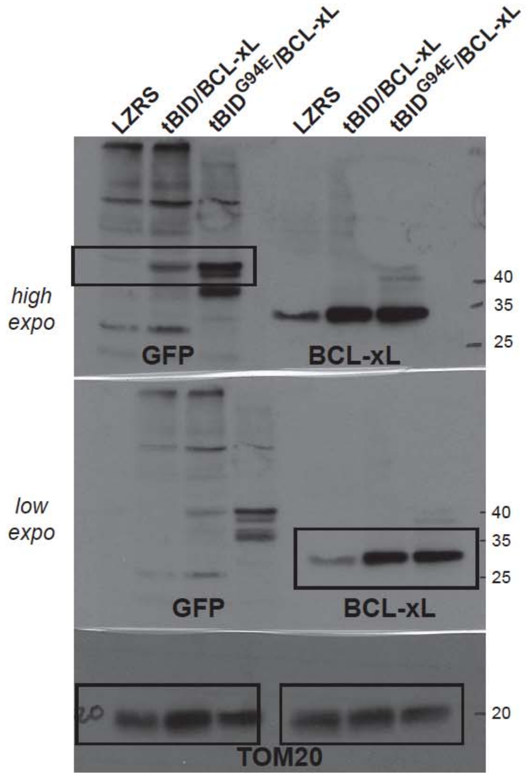

S1f

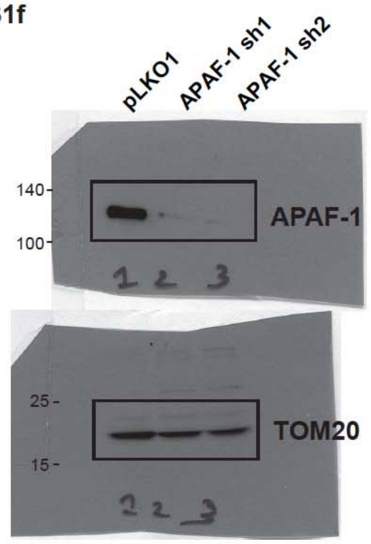

S3a

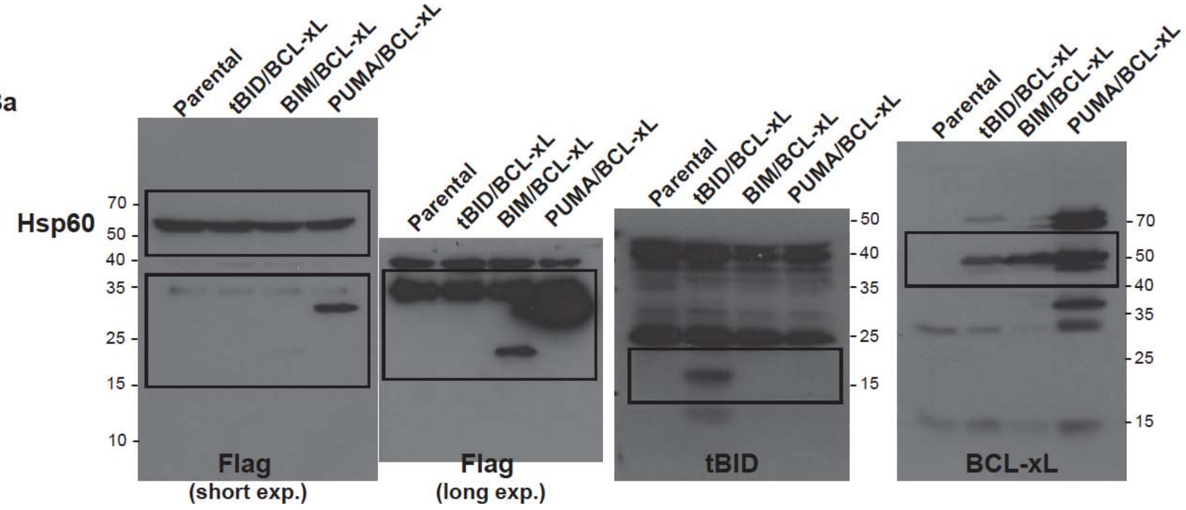
Parental
tBID/BCL-xL
BIM/BCL-xL
PUMA/BCL-xL
Hsp60
70
50
40
35
25
15
10
Flag
(short exp.)
Flag
(long exp.)
tBID
BCL-xL

S3b

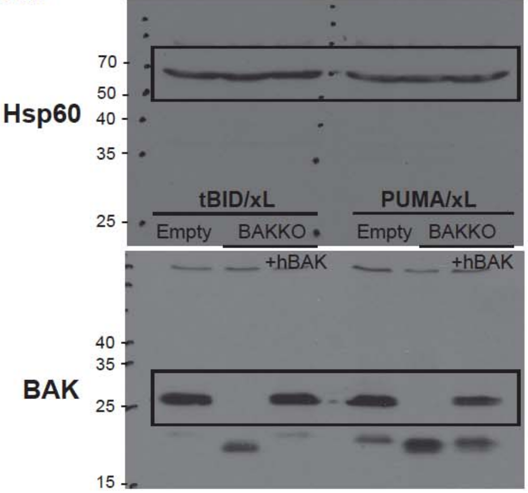
Hsp60
70
50
40
35
25
tBID/xL
PUMA/xL
Empty
BAKKO
+hBAK
BAK
15

S3d

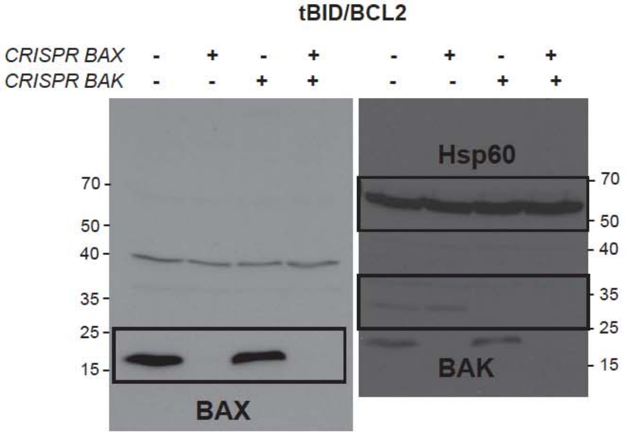
tBID/BCL2
CRISPR BAX
CRISPR BAK
Hsp60
70
50
40
35
25
15
BAX
BAK

S3c

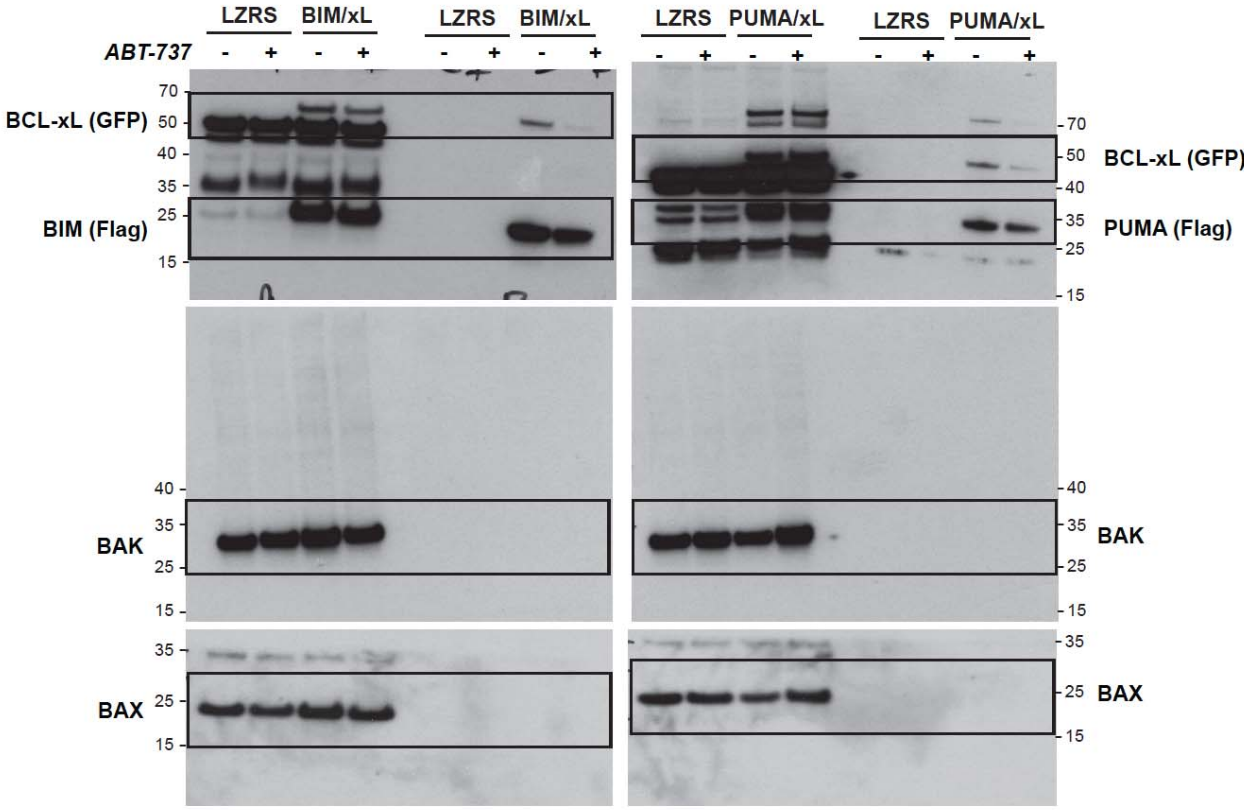
LZRS
BIM/xL
LZRS
BIM/xL
LZRS
PUMA/xL
LZRS
PUMA/xL
ABT-737
BCL-xL (GFP)
BIM (Flag)
PUMA (Flag)
BAK
BAX
70
50
40
35
25
15

S3e

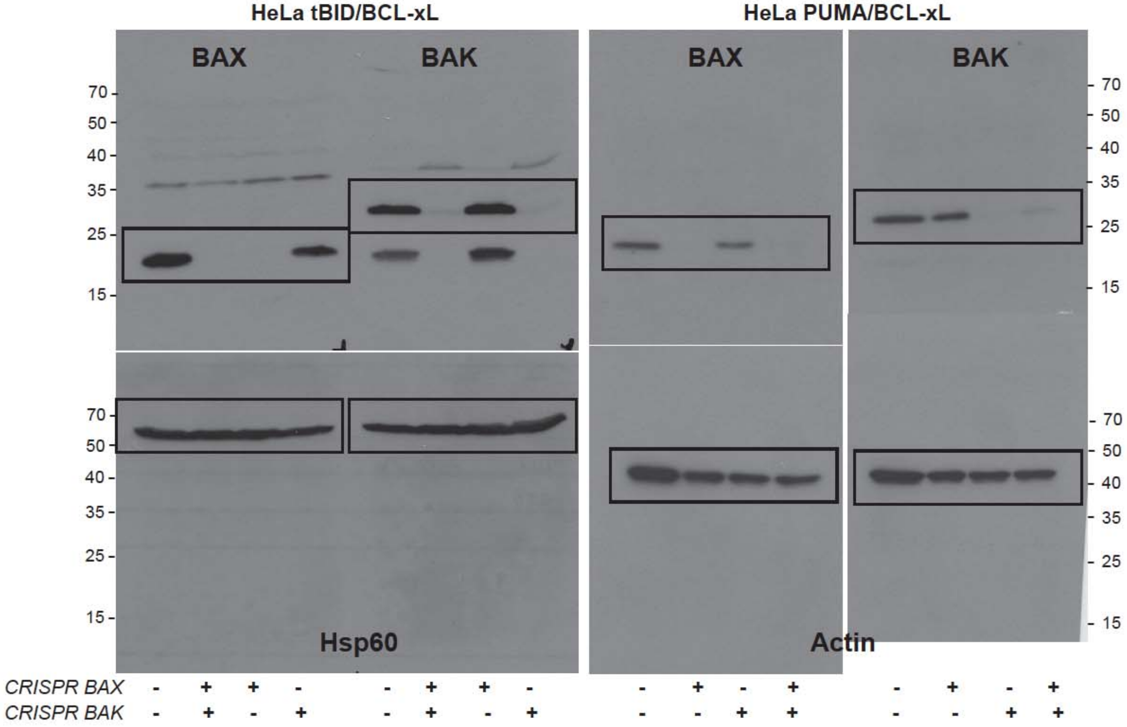
HeLa tBID/BCL-xL
HeLa PUMA/BCL-xL
BAX
BAK
BAX
BAK
Hsp60
Actin
CRISPR BAX
CRISPR BAK
70
50
40
35
25
15

S3f

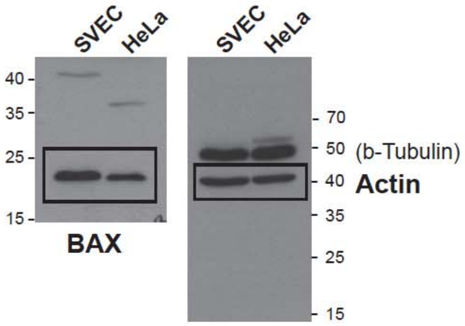
SVEC
HeLa
SVEC
HeLa
40
35
25
15
BAX
70
50
(b-Tubulin)
40
Actin
35
25
15

S3g

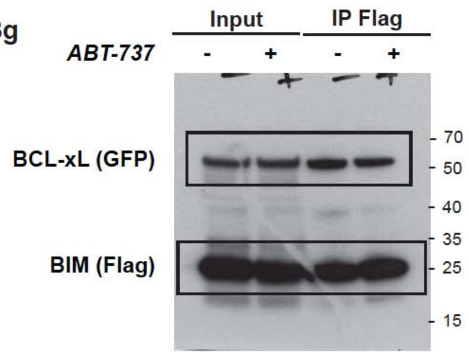
Input
IP Flag
ABT-737
-
+
-
+
BCL-xL (GFP)
BIM (Flag)
70
50
40
35
25
15

S4

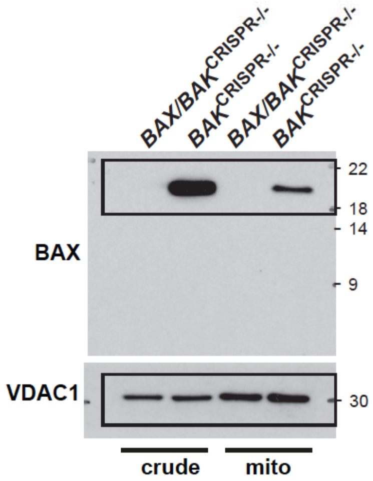
BAX/BAK CRISPR-/-
BAK CRISPR-/-
BAX/BAK CRISPR-/-
BAK CRISPR-/-
22
18
14
9
BAX
VDAC1
30
crude
mito

S5a

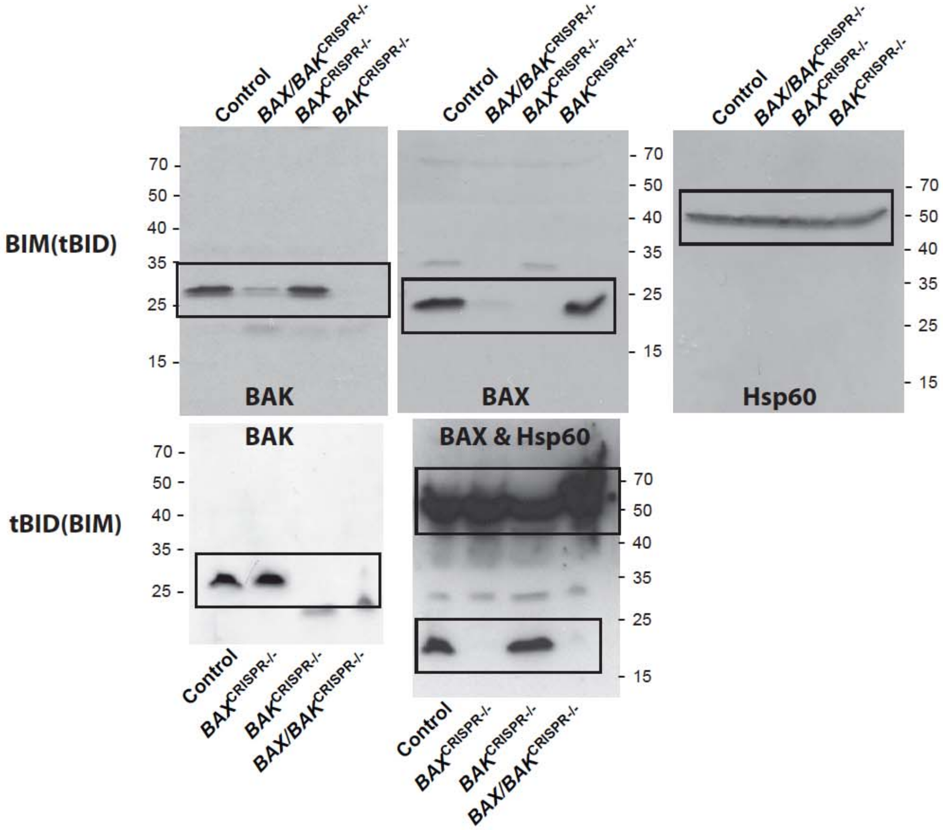

BIM(tBID)
tBID(BIM)
Control
BAX/BAK^CRISPR-/-
BAX^CRISPR-/-
BAK^CRISPR-/-
BAK
BAX
Hsp60
BAX & Hsp60
70
50
40
35
25
15

S5b

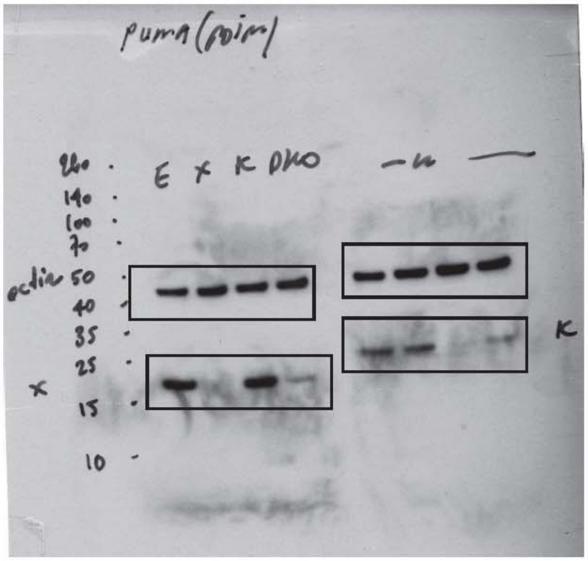

..

Supplementary Movies

Supplementary Movie 1

SVEC cells stably expressing eGFP-tBID 2A BCL-xL were treated with 10μM ABT-737 and analysed for cell viability by SYTOX Green exclusion and IncuCyte cell imaging every 5 minutes for 21 hours.

Supplementary Movie 2

SVEC cells stably expressing eGFP-tBID 2A BCL-xL were treated with 10μM ABT-737 and analysed for cell viability by Annexin V staining (purple). Images were acquired on a Nikon A1R confocal instrument every 5 minutes for 1.5 hour.

Supplementary Movie 3

SVEC cells stably expressing eGFP-tBID 2A BCL-xL were transfected with SMAC-mCherry. Following treatment with 10μM ABT-737, images were acquired on a Nikon A1R confocal instrument every 5 minutes for 1 hour.
